# Novel CAR T therapy is a ray of hope in the treatment of seriously ill AML patients

**DOI:** 10.1186/s13287-021-02420-8

**Published:** 2021-08-20

**Authors:** Faroogh Marofi, Heshu Sulaiman Rahman, Zaid Mahdi Jaber Al-Obaidi, Abduladheem Turki Jalil, Walid Kamal Abdelbasset, Wanich Suksatan, Aleksei Evgenievich Dorofeev, Navid Shomali, Max Stanley Chartrand, Yashwant Pathak, Ali Hassanzadeh, Behzad Baradaran, Majid Ahmadi, Hossein Saeedi, Safa Tahmasebi, Mostafa Jarahian

**Affiliations:** 1 Immunology Research Center, Tabriz University of Medical Sciences, Tabriz, Iran; 2 College of Medicine, University of Sulaimani, Sulaimaniyah, Iraq; 3 Department of Medical Laboratory Sciences, Komar University of Science and Technology, Chaq-Chaq Qularaise, Sulaimaniyah, Iraq; 4 Department of Pharmaceutical Chemistry, College of Pharmacy, University of Alkafeel, Najaf, 54001 Iraq; 5 Department of Chemistry and Biochemistry, College of Medicine, University of Kerbala, Karbala, 56001 Iraq; 6 Faculty of Biology and Ecology, Yanka Kupala State University of Grodno, Grodno, Belarus; 7 Department of Health and Rehabilitation Sciences, College of Applied Medical Sciences, Prince Sattam bin Abdulaziz University, Al Kharj, Saudi Arabia; 8 Department of Physical Therapy, Kasr Al-Aini Hospital, Cairo University, Giza, Egypt; 9 Faculty of Nursing, HRH Princess Chulabhorn College of Medical Science, Chulabhorn Royal Academy, Bangkok, 10210 Thailand; 10 Sechenov First Moscow State Medical University, Moscow, Russia; 11 DigiCare Behavioral Research, Casa Grande, AZ USA; 12 Taneja College of Pharmacy, University of South Florida, Tampa, FL USA; 13 Department of Pharmaceutics, Faculty of Pharmacy, Airlangga University, Surabaya, Indonesia; 14 Stem Cell Research Center, Tabriz University of Medical Sciences, Tabriz, Iran; 15 Department of Immunology, School of Public Health, Tehran University of Medical Sciences, Tehran, Iran; 16 German Cancer Research Center, Toxicology and Chemotherapy, No. 2, Floor 4 Unit (G401), 69120 Heidelberg, Germany

**Keywords:** Acute myeloid leukemia, Adoptive cell therapy, Chimeric antigen receptor T cells, Hematological malignancy, Target antigen

## Abstract

Acute myeloid leukemia (AML) is a serious, life-threatening, and hardly curable hematological malignancy that affects the myeloid cell progenies and challenges patients of all ages but mostly occurs in adults. Although several therapies are available including chemotherapy, allogeneic hematopoietic stem cell transplantation (alloHSCT), and receptor-antagonist drugs, the 5-year survival of patients is quietly disappointing, less than 30%. alloHSCT is the major curative approach for AML with promising results but the treatment has severe adverse effects such as graft-versus-host disease (GVHD). Therefore, as an alternative, more efficient and less harmful immunotherapy-based approaches such as the adoptive transferring T cell therapy are in development for the treatment of AML. As such, chimeric antigen receptor (CAR) T cells are engineered T cells which have been developed in recent years as a breakthrough in cancer therapy. Interestingly, CAR T cells are effective against both solid tumors and hematological cancers such as AML. Gradually, CAR T cell therapy found its way into cancer therapy and was widely used for the treatment of hematologic malignancies with successful results particularly with somewhat better results in hematological cancer in comparison to solid tumors. The AML is generally fatal, therapy-resistant, and sometimes refractory disease with a disappointing low survival rate and weak prognosis. The 5-year survival rate for AML is only about 30%. However, the survival rate seems to be age-dependent. Novel CAR T cell therapy is a light at the end of the tunnel. The CD19 is an important target antigen in AML and lymphoma and the CAR T cells are engineered to target the CD19. In addition, a lot of research goes on the discovery of novel target antigens with therapeutic efficacy and utilizable for generating CAR T cells against various types of cancers. In recent years, many pieces of research on screening and identification of novel AML antigen targets with the goal of generation of effective anti-cancer CAR T cells have led to new therapies with strong cytotoxicity against cancerous cells and impressive clinical outcomes. Also, more recently, an improved version of CAR T cells which were called modified or smartly reprogrammed CAR T cells has been designed with less unwelcome effects, less toxicity against normal cells, more safety, more specificity, longer persistence, and proliferation capability. The purpose of this review is to discuss and explain the most recent advances in CAR T cell-based therapies targeting AML antigens and review the results of preclinical and clinical trials. Moreover, we will criticize the clinical challenges, side effects, and the different strategies for CAR T cell therapy.

## Introduction

AML is a severe hematological malignancy, which although commonly occurs among adults, also is ranked second most common childhood leukemia. AML is an aggressive and heterogeneous cancer that affects the blood and bone marrow. It is characterized by deviated function and proliferation of immature white blood cells (WBCs). At this type of malignancy, the hematopoietic stem cells (HSCs) are persuaded to proliferate in an uncontrollable manner followed by the overproduction of immature WBCs [1, 2]. To date, a wide variety of therapeutic strategies have been developed for AML including chemotherapy, target therapy, and immunotherapy-based treatments [3].

The main choice of treatment to reach a full remission and hampered chance of relapse is chemotherapy which itself is accompanied by alloHSCT. However, the prognosis of refractory/relapsed AML has remained poor and its 5-year survival rate is around 27% [2, 3]. Unfortunately, the treatment strategy comprised of chemotherapy and alloHSCT suffers from some serious disadvantages such as severe and long-term toxic effects on healthy/non-cancerous tissues/organs, restrictions in eliminating the leukemic stem cells, and failed outcomes due to weakness in targeted therapy [4, 5].

Some immunotherapy approaches have emerged with effective applications and interestingly they achieved more successful results when administered in hematological malignancies and solid tumors [6]. Over the last decade, it was illustrated that immunotherapy-based strategies are attractive for AML patients resistant to chemotherapy. These novel therapies can specifically target the antigens on leukemic stem cells and leukemic blasts so less toxicity is achieved by using the therapy [7].

Vaccine therapy, monoclonal antibodies, checkpoint inhibitors, stem cell transplantation, and CAR T cell therapy are some of the immunotherapy techniques with promising outcomes in the treatment of AML [7].

Several new therapeutic agents based on antibodies have been developed targeting key molecules of AML. For instance, gemtuzumab ozogamicin (Mylotarg®; Pfizer) is a humanized mAb linked to an anti-tumor antibiotic, calicheamicin, and so is considered as an antibiotic-antibody conjugate. It was approved by the United States Food and Drug Administration (FDA) for treatment of relapsed AML in adults and children with confident therapeutic results [8]. The ADC treatment alongside other antibody-based therapies such as bispecific T cell engager (BiTE) antibodies achieved successful outcomes.

Therefore, recent advances provide an impressive breakthrough in the treatment of hematological malignancies (e.g., AML). Engineered CAR T cells are one of the novel therapy methods and also a kind of adoptive cell therapy (ACT) [9, 10]. A special type of engineered autologous T cell expressing recombinant receptors specific for target tumor antigens is called a CAR T cell. CAR is composed of the antigen-binding region and functional part of T cell [11]. CAR T cells have many advantages including MHC-independent antigen recognition, acting more specific than TCR, programmable to recognize any tumor antigens, higher proliferation, longer persistence, manageable cytotoxicity capacity, and the capability of preventing tumor escape; therefore, it is a superior therapeutic choice in cancer treatment [12, 13]. For instance, the anti-CD19 CAR T cell is one of the commonly used CAR T cells which has exhibited strong and long-lasting anti-tumor activity in acute lymphocytic leukemia (ALL). It is the first CAR T cell approved by U.S. FDA [14].

In this review, we will discuss the current therapeutic approaches for AML with a special focus on the CAR T cell therapy, structure, generation, and function of CARs, trials on the CAR T cells for the treatment of AML, possible challenges and toxicity of CARs, as well as the suggestions for improving the CAR T cells’ safety and efficacy and for decreasing the toxicity.

## Therapeutic approaches for AML

### Chemotherapy

Chemotherapy is an aggressive approach performed by the injection of anti-cancer (cytotoxic) drugs to kill growing cells in the body. It has been shown that chemotherapeutic drugs are persistent in the bloodstream, so can affect the organs, tissues, and immune cells. Although chemotherapy kills rapidly dividing cells, its harmful effects on healthy tissues are inevitable. The most important side effect of chemotherapy is the weakening of the immune system, which reduces the ability to fight infection, bleeding, and fatigue. Chemotherapy is the main treatment for AML. Common methods of chemotherapy include intravenous (IV) injections. Chemotherapy for AML is divided into three stages: induction, post-remission, and consolidation. The chemical drugs most commonly used to treat AML are a combination of cytarabine with daunorubicin or idarubicin. However, some drugs such as fludarabine, 6-thioguanine (6-TG), methotrexate (MTX), and azacitidine can be used [15–17].

### Antigen targeted therapy

#### Hedgehog (Hh) signaling pathway

Hh is a key pathway required for the survival of leukemia stem cells which can be disturbed by specific inhibitors. Glasdegib, an oral Hh pathway suppressor, has demonstrated a strong suppression effect on the proliferation of AML cells [18, 19]. The promising responses and good tolerability of Glasdegib were observed in the phase I trial study [20].

#### Fms-like tyrosine kinase 3 (FLT3)

FLT3 is a transmembrane tyrosine kinase and its mutant forms are mostly overexpressed in AML [21]. Midostaurin is one of the important preventing agents against FLT3, which has shown beneficial effects on repressing the AML and increasing the survival rate especially when it is used along with chemotherapy agents [22].

#### Isocitrate dehydrogenase 1,2 (IDH1/2)

Ivosidenib and Enasidenib are other chemical agents used to restrain the functionality of IDH1 and IDH2 mutant forms (in cancerous cells), respectively. The mutant forms cause a cascade of harmful intracellular changes such as enhanced 2-hydroxyglutarate production, an oncometabolite that inhibits the enzymes with an important role in histone methylation. The subsequent changes in methylation processes cause transcriptional dysregulation and therefore resulted in the promotion of proliferation and blockade of differentiation of myeloid cells. The drugs targeting IDH1 and IDH2 reverse these harmful changes and therefore cause therapeutic effects in AML patients [23].

#### B cell leukemia/lymphoma-2 (BCL2) and P53

Survival of leukemic blasts (myeloblasts) is mainly associated with the presence of anti-apoptotic proteins termed B cell leukemia/lymphoma-2 (BCL2). Venetoclax is a BCL-2 homology 3 (BH3)-mimetic compound that returns the apoptosis capability in target BCL-2-mutant cells and therefore it can induce apoptosis in targeted cancerous cells [24, 25]. In addition, it was known that the overexpressed mutant form of P53 in AML cells can cause cancer development. APR-246 is a novel therapeutic molecule and one of the powerful blockers of P53 mutant form and can mediate the P53 refolding and reactivation to induce the apoptosis/cell cycle arrest in tumor cells [26].

#### E-selectin

E-selectin is an overexpressed cell adhesion molecule on leukemic blasts. Uproleselan (GMI-1271) is an E-selectin blocker that can increase the anti-cancer chemotherapy response against AML [27].

#### Polo-like kinase-1 (PLK1)

PLK1 is a key mitosis regulator and helps the cell for the right passage through mitosis processes. It is thought that PLK1 has a role in DNA modification and replication procedures. Volasertib and Rigosertib are important suppressors of PLK1 and developed for the treatment of AML [28, 29].

#### Cyclin-dependent kinases (CDKs)

CDKs, the members of the protein kinases family, are also important target molecules in AML. CDKs have critical roles in cell cycle regulation, cell differentiation, mRNA preparation, and transcription regulation. CHK1 protein kinase overexpression in AML is correlated with limited responses to chemotherapy and a shorter duration of survival. Prexasertib is a CHK1 inhibitory agent that can restrict the replication processes [30]. Moreover, FDA granted palbociclib as the CDK4/6 inhibitory agent for the treatment of breast cancer. It has a good potential to be used as a therapy in other cancers such as AML [31].

### Vaccine therapy

A vaccine is a prominent example of active immunotherapy which can trigger the immune system responses against a specific antigen. Immune responses can be elicited by various types of vaccines such as dendritic cell (DC) vaccines, peptide-antigen vaccines, and DNA vaccines. For AML, Wilms’ Tumor-1 (WT-1) antigen was used to generate a well tolerable vaccine, which can induce the antigen-specific T cell responses and elicit a moderate but acceptable level of therapeutic responses [32]. Moreover, OCV-501, an HLA peptide derived from WT-1 protein was used to design an antigen-based vaccine for AML. It was evaluated in phase 2 clinical trial and the treatment was well tolerated by elderly AML patients [33]. In addition, various DC vaccines have been developed for the treatment of AML. One of the DC vaccines emanated from AML cell lines was called DCP-001 and was evaluated in a phase I clinical trial study [34]. Also, another DC vaccine which is comprised of WT1 mRNA-electroporated DCs was evaluated against AML in a phase II trial study. The vaccine decreased the commonly occurred relapse during the remission period after AML chemotherapy [35]. Also, another DC vaccine was developed by fusing patient-derived AML cells and autologous dendritic cells, making a hybridoma capable of effectively stimulating the immune system and acts as a personalized anti-cancer therapy. The vaccine was assessed in a third phase clinical trial [36].

### Monoclonal antibody therapy

Various monoclonal antibodies (mAbs) targeting different antigens of tumor cells are widely used as the immunotherapy approaches for AML and other types of cancer. As an example, an anti-CD33 mAb called gemtuzumab was fused to an anti-tumor antibiotic, calicheamicin; a complex comprised of gemtuzumab and calicheamicin, the conjugate, was called gemtuzumab ozogamicin [37]. This complex has been also approved and administered for non-chemo-eligible relapsed AML patients, and it showed good toxicity against leukemic cells. Moreover, other trial studies have been conducted to appraise the gemtuzumab efficacy when administered simultaneously (as combination therapy) with other chemotherapy drugs like decitabine (NCT00882102), cytarabine (NCT02473146), and azacitidine [38]. Another example of antibody-drug conjugate therapy is the vadastuximab talirine, an anti-CD33 mAb conjugated to pyrrolobenzodiazepine, a highly potent DNA binding agent for the treatment of AML [39]. Moreover, there are also some other mAbs under development such as CSL360, IMG632, and SGN-CD123A which target the CD123, an overexpressed antigen in AML cells [40–42].

### Bispecific antibody therapy

Bispecific antibodies (BAB) composed of two distinct variable domains, one specific for a tumor antigen and the other specific for a CD3 receptor on the T cell, have been developed in recent years with some superiority in comparison to monospecific antibodies. BABs can detect the tumor cell and simultaneously activate the killing mechanisms in T cells by engaging them towards the detected tumor cell. An anti-CD3/CD33 bispecific antibody (AMG330) and an anti-CD3/CD123 antibody named flotetuzumab are two good examples of bispecific antibodies, both evaluated for the treatment of AML. Compared to old-fashioned mAbs, BABs have some advantages such as increased safety, more efficacy in tumor cell killing, and reduced exhaustion of the immune cells which is particularly characterized by deletion or inhibited functions of T cells [43, 44].

### Checkpoint inhibitors

Checkpoint inhibitors are used to prevent the negative modulation of the immune system. Checkpoints are proteins/receptors on immune cells particularly T cells and in the case of challenges with the tumor cells, they send an “off” signal to T cell and prevent initiation of killing mechanisms. The checkpoint proteins keep the T cells in check and prevent the overactivation of the immune system. Also, there are partner proteins on tumor cells that can bond to checkpoints and make the T cells deactivated, so the tumor cells can escape from immune system anti-tumor responses. CTLA4 and PD-1 are overexpressed checkpoints in AML patients. These checkpoints have been the subject of the development of novel mAb-based therapies. Currently, there are two mAbs named ipilimumab and nivolumab blocking the checkpoints CTLA4 and PD-1, respectively [45, 46]. PD-1 blocking along with adoptive cell therapy have shown effective anti-tumor responses. Furthermore, higher anti-tumor toxicity was reported in combinational therapies such as nivolumab-ipilimumab and azacitidine-nivolumab [47, 48]. There is also an approach in clinical trials for AML by which affect T cell by PD1 inhibitors such as nivolumab and pembrolizumab along with macrophages by targeting CD47 such as magrolimab. This approach along with hypomethylating agent administration has been under clinical investigations [49].

### Allogeneic hematopoietic stem cell transplantation (alloHSCT)

alloHSCT is one of the beneficial and curative post-remission approaches which is used in various types of cancers particularly hematological malignancies like AML. alloHSCT is done by collecting and transplanting the matching stem cells from a donor into the patient. To completely eradicate tumor cells, patients receive alloHSCT after undergoing a conditioning therapeutic regimen such as chemotherapy or radiation therapy. Then, over the engraftment process, the production of new blood cells is reestablished and restarted by transplanted stem cells. HLA differences between the donor and the recipient can activate the immune system response against the cells, so the closer the tissue “match” between the donor and recipient, the more likely they are to “take” the transplant cells. For most patients with recurrent AML, allogeneic HSCT is preferable to autologous HSCT, for returning sick cells to a patient after treatment may mean returning some leukemia cells to them. Donor cells are more beneficial because of GVL. In this regard, donors’ immune cells recognize and attack to remaining leukemia as soon as injected, which does not happen in autologous HSCT [50, 51]. Clinical investigations revealed a significant survival rate among AML patients after receiving alloHSCT. However, there are some drawbacks or side effects when using the alloHSCT method such as graft-versus-host disease (GVHD), relapse, increased mortality, and infection [52–54]. GVHD is one of the most serious complications of allogeneic HSCTs which happens when the donor’s immune system attacks the recipients’ tissues. Symptoms are including skin rashes, nausea, diarrhea, and jaundice [55]. Although allogeneic HSCT usually needs a younger and healthy candidate, alloHSCT-induced graft versus leukemia (GVL) has been shown to increase disease-free survival in elderly patients with AML. Currently, 22% of allogeneic HSCT procedure have been done on patients older than 60 years; however, alloHSCT has been performed on only 6% of AML patients over the age of 60 in the USA, indicating that hematology units are reluctant to consider an allogeneic HSCT as a treatment option for patients with AML [56].

## CAR T cell therapy

### CAR T cell structure

ACT is a novel and potent immunotherapy-based technique with encouraging results in the treatment of cancer. Also, CAR immune cells particularly T cell, which is generated by genetically engineering T cells, is one of the ACT approaches with promising results. The first proposal for using CARs was presented by Eshhar et al. in 1989 to direct the T cells to target the specific antigens [11]. CAR T cell therapy is a great breakthrough inefficient therapy of blood cancer and even solid tumors particularly to prevent relapse [57, 58]. CAR is expressed as a recombinant receptor composed of the antigen-binding region (ScFv) and a signaling/empowering part on the patient’s T cells. CARs are designed by using part of mAbs and can recognize only one specific antigen epitope. Therefore, CAR T cell engineering is implemented first by isolation of patients’ T cells, next by incorporating the CARs into the T cells, expansion of newly generated CAR T cells, and then re-infusion of newly engineered cells into the patient’s body [59, 60].

**Fig. 1:**
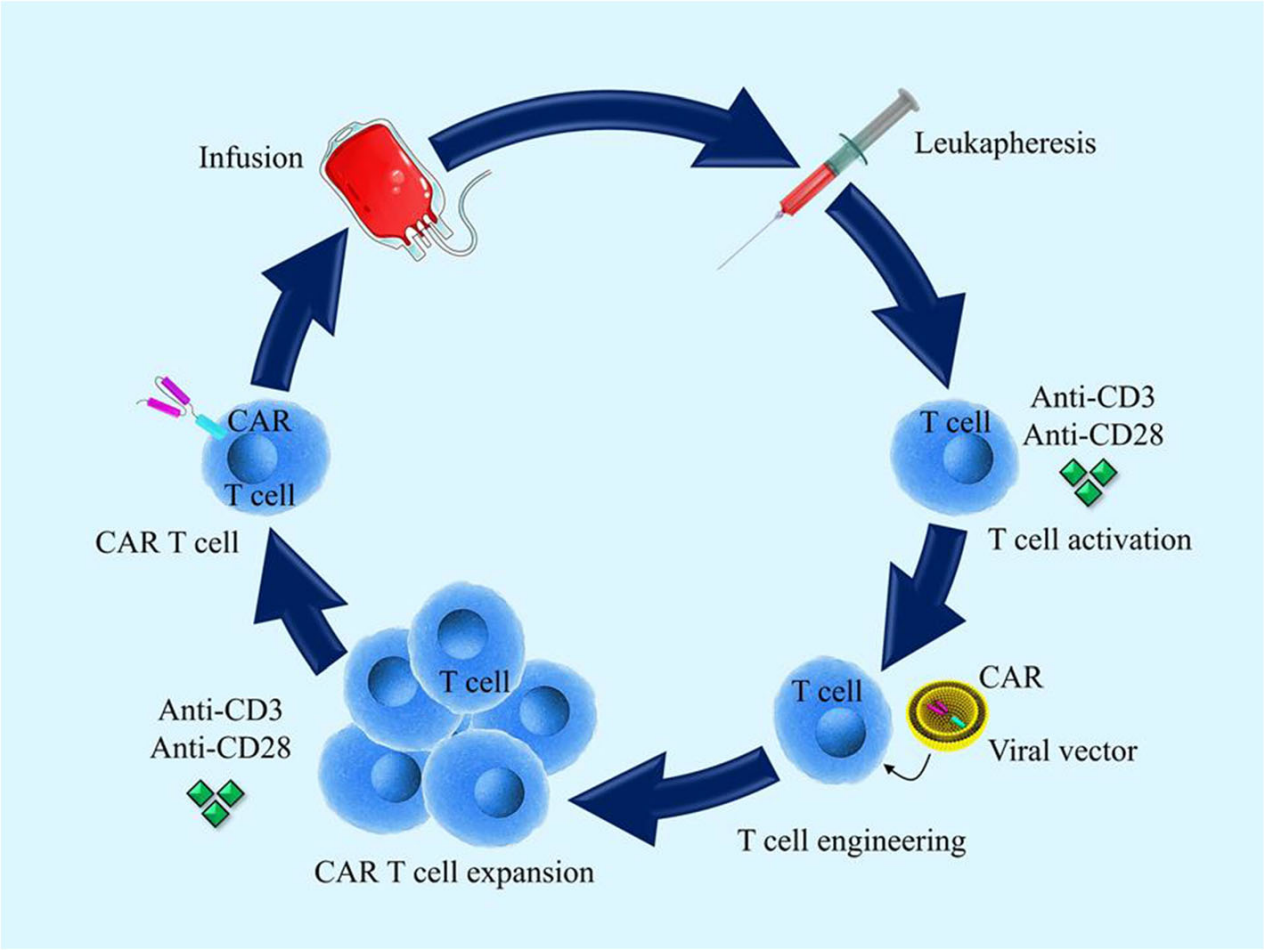
Isolation, engineering, proliferation, and administration of CAR T cells. In the first stage, T cells are isolated from patients through leukapheresis and then harvested and activated. After that, the engineering and construction of the CARs are done in T cells. After the proliferation of the produced CAR T cells, the cells are re-injected into patients

A CAR T cell construct is composed of four main parts as follows (Fig. 1): (1) an antigen recognizing extracellular domain called ScFv which itself is comprised of light and heavy variable chain of a monoclonal antibody. (2) Spacer or hinge region which provides flexibility and is usually made of either IgG-based part such as CH2, CH3, CH2CH3, or even hingeless or Ig-based hinges from naive T cell molecules such as CD8 or CD28. The ScFv and hinge together make the ectodomain part of CAR. (3) Transmembrane part of CAR is placed through the membrane and its structure is usually derived from CD3-ζ, CD4, CD8, or CD28 molecules. (4) Intracellular signaling domain is mainly the cytoplasmic CD3-ζ domain although it can be accompanied by some co-stimulatory molecules such as CD28, 4-1BB, or OX40. Moreover, some other immunoreceptor tyrosine-based activation motifs (ITAMs) such as the Fc receptor for IgE-γ domain have been evaluated as the intracellular activating domain but with less efficacy compared to CD3-ζ [61, 62]. The transmembrane domain and intracellular domain together form the endodomain of CAR. To enforce the naive T cell to generate CARs, CAR transgene is transferred into the naive T cells via various methods such as viral vectors [63], gene-editing techniques [64], mRNA electroporation [65], and liposomes [66].

### The process of CAR T cell development and its various generations

**Fig. 2:**
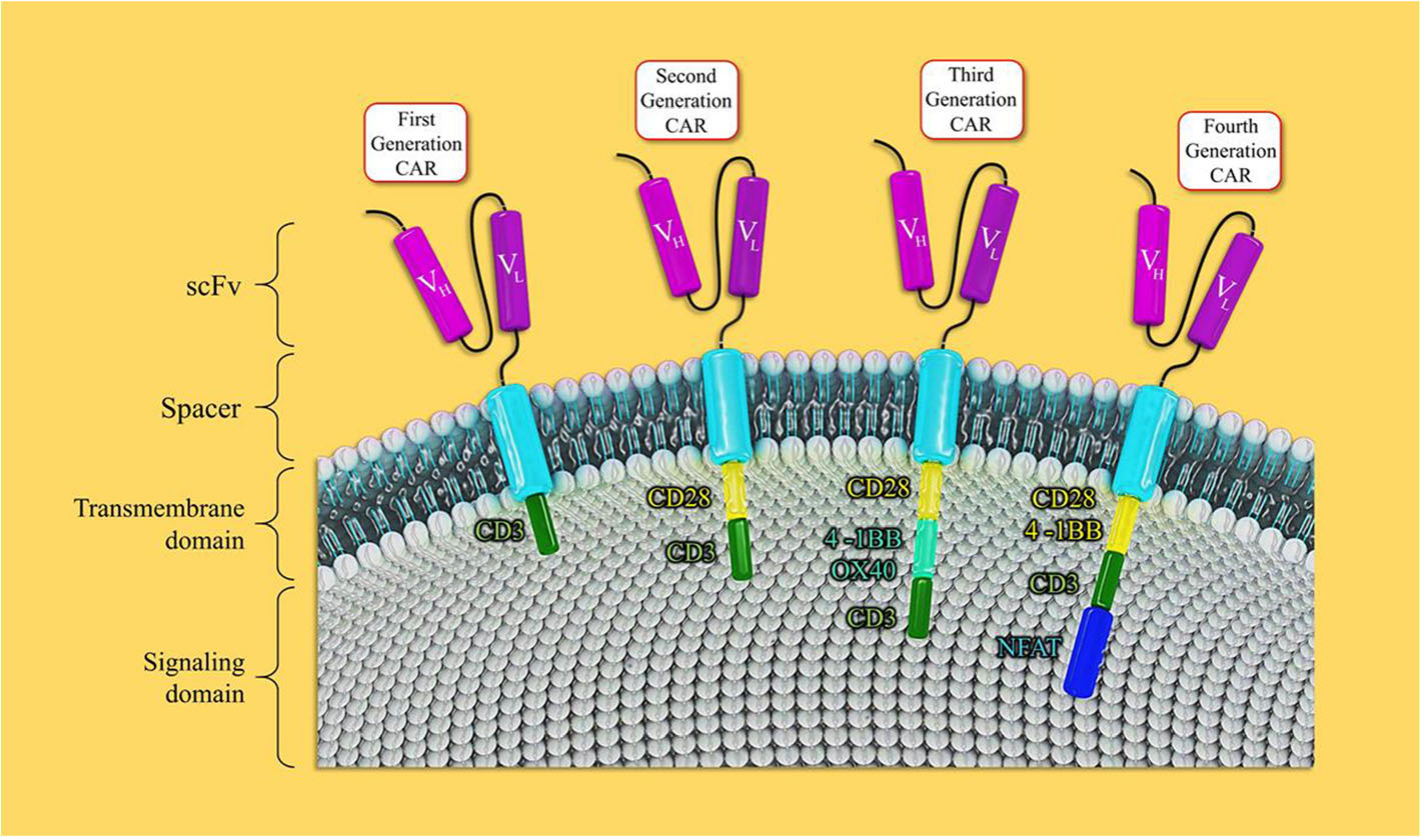
Different generations of CAR T cells. CAR signaling and applications are updated with each new generation. The first generation consists of an intracellular domain (CD3ζ chain), the second generation of CAR T cells has been composed of the addition of CD28 to CD3 as co-stimulatory molecules. In the third generation of CAR T cells, OX-40, a second co-stimulatory molecule, has been added. In the fourth generation, NFAT has been added to the induction of cassette containing the IL-12 gene promoter. NFAT, nuclear factor of activated T cells

Different types of CAR T cells have been developed over time in four generations (Fig. 2). They were distinguished mainly by the variations in co-stimulatory molecules. So, CAR generations are distinguished by enhanced persistence, proliferation, and killing activity. All generations comprise the scFv region and CD3ζ intracellular signaling domain as the base parts. Second generation has also one co-stimulatory domain and the 3rd generation has two co-stimulatory domains in addition to base parts [12]. Fourth generation of CAR T cells termed T cell Redirected for Universal Cytokine Killing (TRUCKs) is the latest developed generation of CAR T cells with the capability of constitutively releasing cytokines (IL-12,1 L-15,1 L-18, and IL-2) or other biological factors to strengthen the anti-tumor activity. TRUCKs are armored with nuclear transcription factors. The armored with CAR-inducible transgenes capable of encoding various cytokine and mediators [67, 68].

### CAR T cell function

In contrast to the classical T cells with recognizing the antigens by T cell receptor (TCR), chimeric antigen receptor (CAR) in engineered CAR T cells has a role in antigen recognition. As well, CAR T cells can identify broader types of antigens in the independence pathway of MHC. Antigen recognition through ScFv followed by intracellular signaling activates the CAR T cell against target cells. After activation, the CAR T cell specifically exerts its functionality through the secretion of anti-tumor cytokines, perforin, and granzymes into the tumor microenvironment (TME) [69, 70]. Therewith, immune system surveillance, recruitment of other immune cells, tumor cell elimination, and inhibition of tumor relapse can be increased by using CAR T cell as a living drug.

### CAR T cell therapy in hematological malignancies

CAR T cell therapy with hopeful success results has been developed in various hematological malignancies without significant efficacy in solid tumors. Immunosuppressive conditions of the solid tumor microenvironment, antigen heterogeneity, and other related hurdles reduce the CAR T cell efficacy in the context of solid tumor tissue [58]. CAR T cell-based therapies with manageable cytotoxicity and high efficacy are widely utilized in hematological cancers such as acute and chronic forms of leukemia, lymphoma, and multiple myeloma [13].

In hematological cancers, CD19 is a commonly used target molecule in generating CAR T cells. Several done or undergoing clinical studies have been conducted to evaluate the CD19-CAR T cell therapeutic function in relapsed/refractory (R/R) leukemias and lymphomas [71–73]. Encouragingly, U.S. Food and Drug Administration (FDA) has approved CD19-CAR T cell so-called Kymriah (CTL019) due to the efficient therapeutic findings, and safety for adults and children with acute lymphoblastic leukemia (ALL) [14]. Lately, CD19-CAR T cell efficacy has been also validated for R/R diffuse large B cell lymphoma (DLBCL) and R/R follicular lymphoma (FL), which highlighted the remarkable and tolerable anti-tumor function [74–76].

CD22 another overexpressed antigen on B cell cancers can be targeted by the CD22-CAR T cell. The impressive anti-tumor function of CD22-CAR T cell was reported in relapsed cancer after CD19-CAR T cell therapy or in eradicating the ALL tumor cells [77].

Moreover, various studies affirmed the CD20-CAR T cell safety and efficacy in R/R Non-Hodgkin’s lymphoma (NHL), follicular- and mantle cell lymphomas [78, 79]. Additionally, treatment of chronic lymphocytic leukemia (CLL) as another common hematological malignancy was investigated by using the engineered CAR T cell against CD19 or the tyrosine-protein kinase transmembrane receptor [80, 81]. Another useful CAR T cell therapy in B cell malignancies is the anti-κ/λ CAR T cell which has demonstrated the cytotoxicity against malignant B cells without effects on normal cells [82]. Hopefully, CAR T cell therapy has pointed to the encouraging results in multiple myeloma (MM), a bone-marrow-derived refractory hematological malignancy. Surprisingly, in clinical trial studies, anti-CD138 CAR T cells and anti-BCMA CAR T cells designed for MM represented the powerful and well-tolerated activity against tumor cells. Conversely, CD19-CAR T cells in MM revealed a non-significant function against myeloma cells due to the lower expression of CD19 antigen [83, 84].

## CAR T cell therapy and potential targets in AML

In AML, due to the difficult identification of target antigens, CAR T cell therapy is encountered various challenges and no approved study exists related to CAR T cell therapy for AML yet. Therefore, increasing the understanding of the AML microenvironment can be contributed to discovering the enthusiastic and proper target antigens (Table 1). Based on this, several clinical studies have been developed to investigate the CAR T cell therapeutic function against predisposed antigens such as CD7, CD33, CD38, CD44, CD70, CD123, CLL, FLT3l, FRβ, Le-Y, LILRB2, NKG2D, PR1, and WT1. In this review, various types of CAR T cells against candidate antigens have been described (Table 2).

**Table 1:** AML target molecules

Target antigens	Type of molecule	Role	On normal cells	On HSCs	On LSCs	On AML blasts	References
**CD7**	Ig superfamily/ Glycoprotein	B and T cell lymphoid development, transmembrane protein	T, NK cells, and myeloid progenitors	No	Yes	Yes	[85]
**CD33**	The protein of the SIGLEC family	Transmembrane receptor	Progenitor, myeloid, and kupffer cells	Yes	Yes	Yes	[86, 87]
**CD38**	Glycoprotein	Cyclic ADP ribose hydroxylase, a transmembrane protein	B, T, NK cells	No	Yes	Yes	[88, 89]
**CD44v6**	Glycoprotein	Transmembrane protein	Keratinocytes	No	Yes	Yes	[90]
**CD70**	Glycoprotein from the TNF family	Transmembrane protein	T and B cell	No	Yes	Yes	[91, 92]
**CD123**	Type I cytokine receptor of IL-3	IL-3 receptor α subunit	Myeloid progenitors, DC and, basophils	Yes	Yes	Yes	[92–94]
**FLT3**	Type III cytokine receptor	Tyrosine kinase receptor	Neurons, testis	Yes	Yes	Yes	[95, 96]
**CCL1**	Glycoprotein	Transmembrane receptor	Myeloid, lung, epithelial cells	No	Yes	Yes	[97, 98]
**LeY**	Glycosphingolipid (fucosyltransferase)	Blood group Ag	Intestinal epithelial cells	Yes	Yes	Yes	[99, 100]
**FRβ**	Folate-binding protein receptor	Folate delivery	Myeloid cells	No	Yes	Yes	[101]
**LILRB4**	Leukocyte Ig-like Receptor-B family	Inhibitory receptor role in immune tolerance	Monocytes	No	Yes	Yes	[102]
**NKG2D**	C-type lectin-like receptor protein	Activator receptor	NK, NKT, Tγδ, Th, and CTL	No	Yes	Yes	[103, 104]
**PR1**	Proteinase protein	HLA-presented antigens	Neutrophils	No	No	Yes	[105]
**WT1**	Zinc-finger DNA binding protein	Transcription factor	Kidney endometrium and testis cells	No	No	Yes	[106]
**mLPA**	Methyl-lysophosphatidic acid	CD1c-restricted T cell antigen	MO, DC, B and T cells	No	Yes	Yes	1
**IDH1(R132)**	Isocitrate dehydrogenase 1	Glyoxylate bypass, tricarboxylic acid cycle	Hepatocytes, cytotrophoblasts	Yes	Yes	Yes	2.3.4
**IDH2(R140)**	Isocitrate dehydrogenase 2	Glyoxylate bypass, tricarboxylic acid cycle	Distal tubular cells, cytotrophoblasts	Yes	Yes	Yes	5
**NPM1**^**mut**^	Nuclophosmin 1 mutant	Biogenesis of ribosomes, Chaperone, Host-virus interaction	Low cell and tissue type specificity	No	Yes	Yes	6,7
**NOTCH2**	NOTCH signaling molecule isoform 2	Developmental processes	Non-lymphoid progenitor cells, Paneth cells	Yes	Yes	Yes	8
**MUC1**	Glycoprotein	Protective function cell signaling	Surface of most simple epithelia and Treg cells	Yes	Yes	Yes	9, 10
**CD96**	Member of immunoglobulin superfamily	Adhesion of activated T and NK cells	T cells and NK cells	No	Yes	Yes	11
**PRL3**	Protein tyrosine phosphatase type Iva member 3	Reinforcing PI3K/Akt activation	Cardiomyocyte, neutrophil, non-classical monocyte	Yes	Yes	Yes	12, 13
**IL12RB1**	Interleukin 12 receptor beta 1	Cytokine signaling	T cells, Kupffer cells, B cells	Yes	Yes	Yes	11

*Abbreviations*: *AML* acute myeloid leukemia; *HSCs* hematopoietic stem cells; *LSCs* Leukemic stem cells; *Ig* immunoglobulin; *NK* Natural killer cell; *SIGLEC* sialic acid-binding immunoglobulin-like lectin; *ADP* adenosine diphosphate; *TNF* tumor necrosis factor; *FLT3* Fms-like tyrosine kinase 3; *CCL1* c-type lectin-like molecule-1; *LeY* Lewis Y; *FRβ* folate receptor β; *LILRB* leukocyte immunoglobulin-like receptor B4; *NKG2D* Natural killer group 2 D; *PR1* proteinase; *WT1* Wilms Tumor 1; *DC* dendritic cell; *mLPA* methyl-lysophosphatidic acid; *IDH1* isocitrate dehydrogenase 1; *NPM1mut* nuclophosmin 1 mutant; *NOTCH2* NOTCH signaling molecule isoform; *MUC1* mucin1; *PRL3* protein tyrosine phosphatase type Iva member 3; *IL12RB1* interleukin 12 receptor beta 1

**Table 2:** CAR T cell against AML target antigens

Target antigen	Clinical trial ID	Phase	Disease	Institution
**CD33**	NCT03126864	I	R/R AML	University of Texas MD Anderson Cancer Center, Houston, Texas, United States
	NCT02799680	I	R/R AML	Affiliated Hospital of Academy of Military Medical Sciences, Beijing, Beijing, China| Chinese PLA General Hospital, Beijing, Beijing, China
	NCT01864902	I/II	R/R AML	Biotherapeutic Department and Pediatrics Department of Chinese PLA General Hospital, Hematological Department, Affiliated Hospital of Changzhi Medical College, Beijing, Beijing, China
	NCT02944162	I/II	R/R AML	PersonGen BioTherapeutics (Suzhou) Co., Ltd., Suzhou, Jiangsu, China
	NCT03291444	I	R/R AML, MDS; ALL	Zhujiang Hospital, Southern Medical University, Guangzhou, Guangdong, China
	NCT03473457	n.a.	R/R AML	Southern Medical University Zhujiang Hospital, Guangdong, Guangdong, China
	NCT03222674	I/II	AML	Zhujiang Hospital of Southern Medical University, Guangzhou, Guangdong, China|
**CD38**	NCT03291444	I	R/R AML, MDS; ALL	Zhujiang Hospital, Southern Medical University, Guangzhou, Guangdong, China
	NCT03473457	n.a	R/R AML	Southern Medical University Zhujiang Hospital, Guangdong, Guangdong, China
	NCT03222674	I/II	AML	Zhujiang Hospital of Southern Medical University, Guangzhou, Guangdong, China|
**CD123**	NCT03585517	I	AML	Xian Lu, Beijing, China
	NCT03114670	I	Recurred AML after alloHSCT	Fengtai District, Beijing Shi, China
	NCT03556982	I/II	R/R AML	307 Hospital of PLA, Beijing, Beijing, China
	NCT02623582	I	R/R AML	Abramson Cancer Center of the University of Pennsylvania, Philadelphia, Pennsylvania, United States
	NCT02159495	I	R/R AML	City of Hope Medical Center, Duarte, California, United States
	NCT03672851	I	R/R AML	Second Affiliated Hospital of Xi'an Jiaotong University, Xi'an, Shaanxi, China
	NCT03766126	I	R/R AML	University of Pennsylvania, Philadelphia, Pennsylvania, United States
	NCT03291444	I	R/R AML, MDS; ALL	Zhujiang Hospital, Southern Medical University, Guangzhou, Guangdong, China
	NCT03473457	n.a	R/R AML	Southern Medical University Zhujiang Hospital, Guangdong, Guangdong, China
	NCT03796390	I	R/R AML	Hebei Yanda Ludaopei Hospital Langfang, Hebei, China
	NCT03222674	I/II	AML	Zhujiang Hospital of Southern Medical University, Guangzhou, Guangdong, China
**UCART123**	NCT03190278	I	R/R AML	Dana-Farber Cancer Institute Boston, Massachusetts, United States Weill Medical College of Cornell University New York, New York, United States, MD Anderson Cancer Center Houston, Texas, United States
	NCT01864902	I	R/R AML, high-risk AML	Weill Cornell Medical College, New York, New York, United States MD Anderson Cancer Center, Houston, Texas, United States
**CD123/CLL1**	NCT03631576	II/III	R/R AML	Fujian Medical University Union Hospital, Fuzhou, Fujian, China
**CD33/CLL1**	NCT03795779	I	R/R AML, MDS, MPN, CML	The General Hospital of Western Theater Command Chengdu, China Peking University Shenzhen Hospital Shenzhen, China
**CCL1**	NCT03222674	I/II	AML	Zhujiang Hospital of Southern Medical University, Guangzhou, Guangdong, China| Shenzhen Geno-immune Medical Institute, Shenzhen, Guangdong, China| Yunnan Cancer Hospital & The Third Affiliated Hospital of Kunming Medical University & Yunnan Cancer Center, KunMing, Yunnan, China
**Lewis Y**	NCT01716364	I	Myeloma, AML, MDS	Peter MacCallum Cancer Centre, Melbourne, Victoria, Australia
**WT1**	NCT03291444	I	R/R AML, ALL, MDS	Zhujiang Hospital, Southern Medical University, Guangzhou, Guangdong, China
**CD7/NK92**	NCT03018405	I/II	R/R AML	PersonGen BioTherapeutics (Suzhou) Co., Ltd., Suzhou, Jiangsu, China
**NKG2D**	NCT02203825	I	AML, MDS-RAEB, MM	Dana-Farber Cancer Institute, Boston, Massachusetts, United States
	NCT03018405	I/II	R/R AML, AML, Myeloma	Tampa, Florida, United States| Buffalo, New York, United States| Brussels, Belgium|Brussels, Belgium| Ghent, Belgium

*Abbreviations*: *R/R* relapsed/refractory, *AML* acute myeloid leukemia, *ALL* acute lymphoblastic leukemia, *MDS* myelodysplastic syndrome, *CML* chronic myeloid leukemia, *MPN* myeloproliferative neoplasm, *alloHSCT* allogeneic hematopoietic stem cell transplantation, *RAEB* refractory anemia with excess blasts, *MM* multiple myeloma

### CD7-CAR T cell

CD7 is a transmembrane glycoprotein expressed by T cells, NK cells, and cord blood myeloid progenitors with a co-stimulatory role in B and T cell lymphoid development interactions. CD7 is also expressed by leukemic cells like AML (30%) but not by healthy myeloid cells. So, it can be a potential candidate for eradicating tumor cells selectively with no toxicity on normal cells [107]. For engineering CAR T cells, CD7 removal requires limiting the T cell fratricide due to the CD7 expression by T cells [108].

In two studies by Gomes-Silva et al. [85, 108], CD7-CAR T cell was engineered against CD7^+^ tumor cells in a xenograft model of AML. Before CAR T cell generation, the CD7 gene of primary activated T cells was removed by CRISPR/Cas9 strategy. Then, the second generation of CD7-knockout (CD7^KO^) CD28- CD3ζ-CD7-CAR T cell was designed by using the ScFv derived from the anti-CD7 antibody. Astonishingly, findings manifested the high cytolytic effects against AML include notable elimination of primary AML blasts and leukemia colony-forming cells, no toxicity on healthy and erythroid cells, and a high concentration of IFN-γ. Moreover, reduction of tumor burden indicated that CD7-CAR T cell prevents systemic leukemia progression. In consequence, CD7-CAR T cell can be a potent treatment for refractory or relapsed AML.

### CD33-CAR T cell

CD33 as another potential target antigen is a transmembrane protein of the sialic acid-binding immunoglobulin-like lectin (SIGLEC) family with regulatory effect on leukocytes in immune responses. CD33 is expressed on normal progenitor cells, myeloid cells, and more than 90% of AML cells possessing diagnostic and therapeutic capabilities [109].

The recombinant humanized anti-CD33 antibody conjugated calicheamicin so-called gemtuzumab ozogamicin (GO) is used for AML treatment as an only approved drug. Clinical findings of using GO against CD33 illustrated the potential of CD33 as an attractive and possible target antigen for AML [110]. Some in vivo and in vitro studies have shown the sustained substantial anti-tumor activity of CD33-CAR T cell against AML cells, significant tumor eradication, and maintenance of T cell persistence during the cytotoxicity [93, 111, 112].

In a phase I trial study conducted by Wang et al. [113], the safety and efficacy of CD33-CAR T cells were assessed in relapsed and refractory AML patients. They administered a total of 1.12 × 10^9^ autologous T cells with 38% anti-CD33 CAR expression. As a consequence, CD33-CAR T cells displayed notable cytolytic functions against CD33+ blasts such as remarkable tumor degradation in the early stage as well as high maintenance of CAR T cell number and cytotoxicity. However, disease progression, pancytopenia, high fever, and CRS toxicity were observed as adverse effects due to the production of a high level of proinflammatory cytokines. Based upon this, safety measures should be considered to reducing the CD33-CAR T cell.

In a preclinical study, to increase the CAR T cell safety, Kenderian et al. [86] generated the transiently expressed mRNA-modified second-generation CD33-CAR T cell by using the ScFv derived from GO. The study findings disclosed that CD33-CAR T cells can strongly eliminate the human AML cells and myelodysplastic syndrome blasts in mouse xenografts. The reported in vivo and in vitro results of assessing the CD33-CAR T cells were the significant proliferation of CAR T cell, prominent anti-tumor activity, increased level of cytokine production, degranulation, and leukemia burden reduction. Additionally, they suggested that CD33 gene eradication through the CRISPR/Cas9 gene-editing strategy as a CD33 knockout (KO) HSPC contributes to increasing the safety of CAR T cells. In this regard, the results of a study demonstrated that the combined administration of CD33-CAR T cell with CD33 KO HSPC led to targeting the AML cells specifically and reducing the myelotoxicity [114].

### CD38-CAR T cell

CD38 as an AML target antigen is a type II transmembrane glycoprotein expressing on AML blasts but not on normal human hematopoietic stem cells. CD38 The decreased level of CD34 and increased level of CD38 contribute to progenitor cell differentiation [115]. To engineer CD38-CAR T cells against AML cells, the intensity and number of CD38 should be increased due to the 83% responsibility of CD38 expression in AML cells. Based on this, All-trans retinoic acid (ATRA) as a therapeutic factor of acute promyelocytic leukemia (APL) treatment has the ability to inducing CD38 expression on AML cells [115, 116].

In a study, Yoshida and colleges engineered CD38-CAR T cells to target leukemia cells. They showed that ATRA increased the CD38 expression on AML cells, and subsequently, remarkable cytotoxicity of CD38-CAR T cell combined with ATRA was observed in eliminating the tumor cells [88].

### CD44v6-CAR T cell

CD44v6 termed as a variant 6 isoforms of the hyaluronic acid CD44 receptor and class I membrane glycoprotein is overexpressed in AML and other hematological malignancies. CD44v6 expression was also observed on circulating monocytes; however, CD44v6 has shown a low expression on healthy cells and no expression on progenitors, HSCs, and resting T and B cells [117–119]. CD44v6 expression is required for tumor cell growth and is related to poor prognosis of AML and multiple myeloma (MM). So, CD44v6 can be a potential target antigen for AML therapy [90].

In a study by Casucci et al. [90], a second-generation CD44v6-CAR T cell was generated by using the ScFv derived from a humanized anti-CD44v6 antibody to targeting CD44+ AML cells safely and effectively. It has been reported that CD44v6-CAR T cells eradicated the CD44+ tumor cells efficiently along and produced the anti-tumor cytokines. Based on published results, CD44v6-CAR T cells eliminated the tumor cells selectively; however, monocytopenia was observed as only hematologic toxicity of CD44v6-CAR T cell so-called on-target/off-tumor toxicity on circulating monocytes. To overcoming this side effect, nonimmunogenic inducible Caspase 9 (iC9) [120] and thymidine kinase [121] suicide genes co-expressed in CD44v6-CAR T cells were applied to ablating CAR T cell efficiently.

### CD70-CAR T cell

CD70 is a ligand for CD27 identified as a type II transmembrane glycoprotein, a member of the TNF family. CD70 expression is upregulated on APCs and can be expressed on T and B cells which are triggered by stimulatory factors. CD70 expression has been evidenced on AML bulk cells and leukemic stem cells (LSC) but not on normal hematopoietic stem cells (HSCs) [91, 92].

In a preclinical study conducted in the AML xenograft model, a second-generation CD27-CD3ζ-CD70-CAR T cell was generated and validated. Strong capability of proliferation, robust potential cytolytic function in eliminating the tumor cells, increased level of TNF-α and IFN-γ production, and no toxicity on healthy HSCs are the reported applications of the CD70-CAR T cell [92]. These promising outcomes suggest the applicability of CD70 antigen as a proper choice for developing CAR T cell against AML.

### CD123-CAR T cell

Another proper target antigen for AML cell therapy is the IL-3 receptor α subunit (IL3Rα) named CD123. CD123 overexpression has been evidenced on leukemic stem cells (LSCs) and AML blasts and no significant expression on normal hematopoietic stem cells [122]. In a clinical study, anti-CD123 neutralizing monoclonal antibody demonstrated insufficient efficacy against AML [123].

Gill and colleagues engineered a second-generation CD123-CAR T cell to target CD123^+^ AML cells in the xenograft model. Interestingly, strong anti-tumor cytotoxicity, establishing memory T cells, proliferation, persistence, degranulation, and effector cytokine production were reported as results of using CD123-CAR T cell [94].

In a study, Thokala et al. engineered CD123-CAR T cells by using different chains of VL and VH from various CD123 specific monoclonal antibodies (mAbs) instead of only one specific antibody. Findings revealed the CD123-CAR T cell cytotoxicity against CD123^+^ AML cells with no effect on CD123^−^ B cell lymphoma cells and tumor burden reduction. Concerning myelotoxicity of CD123 like CD33, it remains a problem with targeting this antigen for AML therapy. Surprisingly, CAR T cells with ScFv composed of VL and VH from various mAbs presented the low off-tumor toxicity and lysis effect on healthy hematopoietic stem cells compared to CAR T cells with VL and VH chains of only one mAb [124].

### FLT3-CAR T cell

One of the most common mutant genes responsible for about 30% of AML patients is a tyrosine kinase receptor so-called FMS-like tyrosine kinase-3 (FLT3) [125]. Two frequent mutant types of FLT3 are internal tandem duplication (ITD) mutations and tyrosine kinase domain (TKD) mutations accounted for 24% and 7% of AML patients respectively [126, 127]. FLT3 kinase activated by ITD and TKD augments AML progression by initiating the PI3K/Akt, Raf/MEK/ERK, and JAK/STAT5 signaling pathways [128]. In particular, AML patients possessing FLT3 have shown poor prognosis and clinical outcomes. Allogeneic HSCT is the only operative treatment available for FLT3+ AML cases [129]. However, FLT3 as a capable AML target antigen can be targeted by developing new targeted therapy approaches to improve AML treatment.

In a preclinical study [95], a second-generation FLT3-4-1BB-CD3-CAR T cell was engineered by using the anti-human FLT3 antibody-derived ScFv to targeting the FLT3+ tumor cells. According to in vitro investigations, IFN-γ and IL-2 were produced by the FLT3-CAR T cell encountering AML cell lines. Withal, inhibition of leukemia cell proliferation was observed by in vivo evaluation of FLT3-CAR T cell function. Toxicity assessment of FLT3-CAR T cell in xenograft model indicated no significant toxicity to multipotent myeloid progenitors (MMPs) and hematopoietic stem cells (HSCs) along with equivalent toxicity to common myeloid progenitors (CMPs) and granulocyte-macrophage progenitors (GMPs) describing the less hematologic toxicity of FLT3-CAR T cell.

In another preclinical study, Wang et al. [130] engineered FLT3L-4-1BB-CD3ζ-CAR T cell to assessing its functionality against FLT3L+ leukemia cells. As a result, prominent anti-tumor cytotoxicity of CAR T cell against FLT3+ leukemia cells as well as the influential killing of the ITD type FLT3 cells compared to the wild type FLT3 cells was observed by using the CAR T cell. Therewith, AML xenograft model survival was lengthened by the effect of FLT3L CAR T cell. FLT3L-CAR T cell indicated less off-tumor toxicity on healthy progenitor and hematopoietic stem cells.

### CLL1-CAR T cell

C-type lectin-like molecule-1 (CLL1) as an inhibitory receptor is a type II transmembrane glycoprotein that can be a potential candidate for AML CAR T cell therapy. CLL1 expression accounts for 92% in AML cases, which is overexpressed on differentiated myeloid cells and AML blasts. As well, CLL overexpression has been evidenced on leukemic stem cells (LSCs) but not on normal HSCs which proposes the less off-tumor toxicity of CLL1-CAR T cell. Currently, targeting CLL1 by specific monoclonal antibody has revealed an effective therapeutic function against AML along with reducing tumor burden [131, 132].

In a previous study, the third generation of CLL1-CAR T cell composed of anti-CCL1 ScFv fused to CD28, 4-1BB, and CD3 signaling domains was generated. This anti-CCL1 CAR T cell showed the in vitro and in vivo strong functionality against CLL1^+^ tumor cells with a wide production of effector cytokines and chemokines including GM-CSF, TNF-α, IFN-γ, and IL-13 [97].

Moreover, two other related studies reported the results of using second-generation CAR T cells to targeting CLL1^+^ AML cells with sparing normal myeloid precursor cells. Increased persistence and cytotoxicity, efficient tumor elimination, anti-inflammatory cytokine production, and managing tumor relapse were reported as results of CLL1-CAR T cell activity [98, 133]. Importantly, it has been reported that using the inducible caspase 9 (iC9) strategy transduced into T cell along with the CAR gene increased the safety of CLL1-CAR T cells by controlling its function. This approach contributed to inhibiting the CAR T cell overactivity by triggering apoptosis [134].

### LeY-CAR T cell

Lewis Y (LeY) is a carbohydrate tumor-associated antigen related to blood-group members. Its expression has been reported by various epithelial-derived cancers and less expression by healthy tissues [134]. Several studies have noticed the relationship between LeY antigen and progression of solid tumors and hematological malignancies. Due to LeY expression on early myeloid progenitor cells, it can be a proper targeting choice for AML treatment [99]. Hence, to target tumors, T cells like engineered CAR T cells have been developed to redirect against LeY tumor cells.

In a preclinical study, Peinert et al. [99] generated the anti-LeY CAR T cell and then investigated its functionality against AML and MM (multiple myeloma) cells. According to the reported results, engineered LeY-CAR T cells specifically targeted the LeY^+^ cells by producing the IFN-γ.

To conduct clinical trial studies for AML and MM adoptive cell therapy by the CAR T cell, Neeson et al. [135] engineered the CD28-CD3z-LeY CAR T cell by using the autologous T cells based on LeY-T ex vivo transduction and expansion protocol. CD8+ modified T cells showed a cytolytic response against LeY^+^ tumor cells and high-level production of IFN-γ.

### FRβ-CAR T cell

Membrane-associated folate receptor β (FRβ) is a member of proteins bounded to folic acid and has a role in folate transporting. FRβ has an overexpression on some nonepithelial origin malignancies and less expression on most of the normal cells. Its expression was evidenced on myeloid-lineage hematopoietic cells and accounts for expression on 70 % of primary AML cells [136]. Various targeted therapies have been developed as hopeful treatment strategies for malignancies like AML and by targeting the FR family. All-trans retinoic acid (ATRA) elevates the FRβ expression on myeloid leukemia cells but not on negative receptor cells which increased the folate-conjugated drug potential as a treatment in a preclinical study [137, 138].

In a preclinical study [101], the first anti-FRβ-CAR T cell (m909) was engineered to targeting AML cells in vitro and in vivo. The effective cytolytic function of m909 CAR T cell was observed against FRβ + cells in vitro and regression of AML tumor cells in vivo with no toxicity on HPSCs. Moreover, they presented the enhancement functionality of CAR T cells due to the elevated expression of FRβ in the presence of ATRA. In another study [101], a high-affinity (HA) anti-FRβ ScFv-CAR T cell was generated for AML treatment. The strong anti-tumor function and potential lyse myeloid-lineage target cells without toxicity on HSCs were observed through using HA-FRβ-CAR T cells in vitro and in vivo.

Consequently, FRβ can be an encouraging and proper target antigen for AML CAR T cell therapy powerful activity in combination with ATRA and without toxicity on normal cells.

### LILRB4-CAR T cell

Monocytic AML (M5) is a common type of AML in children including 20% of AML cases along with poor outcomes. LILRB4 as a potential target antigen in AML belongs to the family of the leukocyte immunoglobulin-like receptor-B family. LILRB4 expression was reported on healthy monocytic cells and monocytic AML cells in all stages [139].

In a preclinical study, 41BB-CD3ζ-anti-LILRB4-CAR T cell was engineered by using the humanized ScFv to target LILRB4 AML cells specifically. Interestingly, applying powerful cytotoxicity to AML cells in vitro, decrement of xenograft model tumor burden in vivo, and reduction of off-tumor toxicity by sparing normal progenitors and HSPCs were found as results of using LILRB4-CAR T cell [102]. These results have illustrated the potential capacity of LILRB4-CAR T cell therapy in targeting the AML selectively and specifically.

### NKG2D-CAR T cell

Natural killer group 2D (NKG2D) known as an activator receptor with homodimer and hexamer form is expressed on CD8+ T cells, a small amount of CD4+, γδ T cells, NK cell, and NKT cell. NKG2D has a co-stimulatory role on T cells in its native form which depends on TCR antigen recognition. NKG2D has a wide spectrum of ligands that overexpressed encountering DNA damage, infections, and malignant transformation [140, 141]. Broad expression of NKG2D ligands was evidenced in various hematological malignancies such as AML and MM, and solid tumors with no expression on healthy cells [142, 143]. Based on this, NKG2D was identified as a potential target candidate for CAR T cell therapy in clinical applications.

Previous preclinical studies in murine models revealed that NKG2D-CAR T cell led to the induction of host protective immune responses and effective eradication of tumor cells in ovarian cancer, multiple myeloma, and lymphoma. Also, it has been demonstrated that NKG2D along with its various ligand expression could suppress the growth of tumor cells [103, 104, 144].

In a phase I clinical trial study conducted by Baumeiste et al. [145], autologous first-generation NKG2D-CD3ζ-CAR T cell was engineered and validated in AML and myelodysplastic syndrome (MDS) patients. For safety and efficacy evaluation, single intravenous administration of NKG2D-CAR T cell was performed by a dose of 1 × 10^6^ up to 3 × 10^7^ T cells. As a result, in one AML patient, an objective clinical response with a high level of IFN-γ production was reported without any high-grade toxicities. Interestingly, it has been reported there are no long-term toxicities like myeloablation and B cell aplasia, infusion toxicity, autoimmunity, and CRS.

### PR1-CAR T cells

In the intracellular process, leukemia-associated-antigens and neoantigens are presented to T cells by HLA-II molecules. TCR-mimic (TCRm) CARs have been developed to targeting the HLA-presented antigens [10].

One of the important leukemia-associated antigens that arose from Proteinase 3 and neutrophil elastase is Proteinase 1 (PR1), HLA A2-restricted nonameric peptide. These proteinases are normally expressed in neutrophils azurophilic granules and are overexpressed on myeloid leukemia blasts [146]. To targeting PR1+ AML cells, Ma et al. developed the anti-PR1/HLA-A2-second-generation CAR T cell by using the ScFv derived from anti-PR1/HLA-A2 TCR-like antibody so-called h8F4. As a consequence, the PR1-CAR T cell exhibited the effective avidity to the PR1+ target cell and preferentially targeted the human AML cell lines and primary AML blasts with high cytotoxicity in vitro. Also, outcomes of evaluating the CAR T cell off-target toxicity have illustrated that leukemia progenitor cells were preferentially suppressed by anti-PR1/HLA2-2-CAR T cell rather than normal hematopoietic progenitors [105]. The successfully reported findings of the h8F4-CAR suggesting the potential of endogenous self-antigens for targeting by CAR T cell in AML.

### WT1-CAR T cells

Wilms Tumor 1 (WT1) as an oncogenic, zinc-finger transcription factor is another HLA A2-restricted intracellular target antigen in AML. WT1 has an important role in the various cellular processes such as organ development, differentiation, proliferation, and apoptosis. Normally, WT1 has less expression by the bone marrow, kidney, gonads, and spleen; however, its overexpression has been proved in various hematological malignancies like AML and CML, and several solid tumors including glioblastoma, mesothelioma, ovarian cancer, and gastrointestinal cancers. Indeed, the poor prognosis of AML and lymphoid leukemia patients is strongly related to WT1 overexpression of WT1 on tumor cells [147–149].

In a study conducted by Rafiq et al. [106], WT1-CAR T cell as another TCRm CAR was developed and validated by using the HLA-A*02:01-a peptide that arose from WT1 antigen. In practice, WT1^+^/HLA-A*02:01^+^ primary tumor cells or cell lines were distinguished and lysed by WT1-CAR T cell. Based on this, prominent cytotoxicity and significant secretion of IL-2 and IFN-γ were observed against primary AML cells or cell lines.

## CAR T cell improvement strategies, side effects, and challenges

**Fig. 3:**
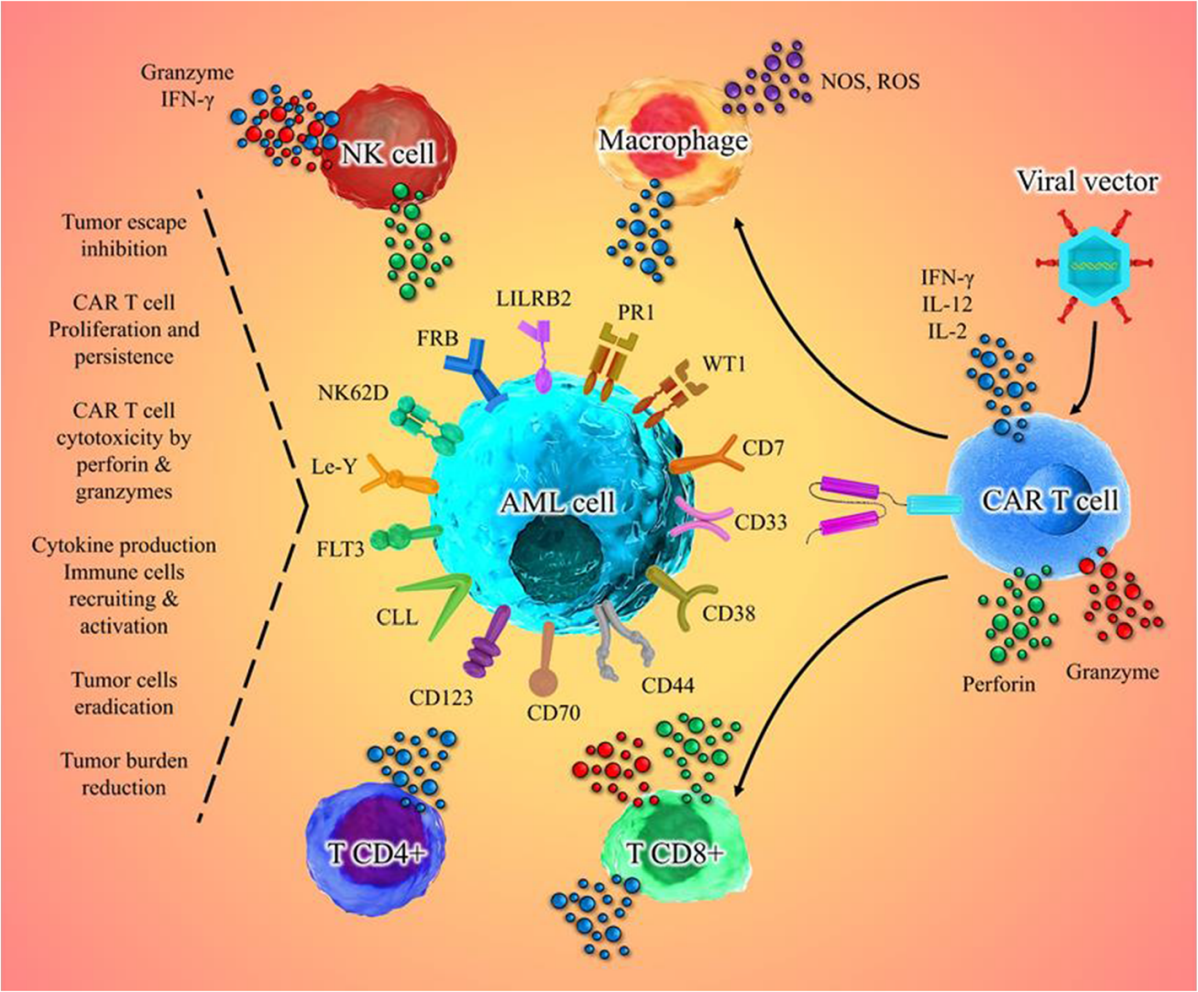
Implications of the association between the CAR T cell and cancer cells in AML. The production of a CAR T cell in patients with AML leads to its identification and binding to TAAs or TSAs on the surface of the cancer cell and has a variety of effects, including activation of macrophages to produce ROS and NOS, activation of NK cells to secrete perforin and granzyme, and secretion of cytokines that strengthen the body’s immune system, which eventually kills tumor cells

Despite the more beneficial effects of CAR T cells, their proliferation, persistence, and anti-tumor functions may decrease encountering some challenges in hematological malignancies or solid tumors (Fig. 3).

### Improvement strategies

#### CAR T cell delivery

Intravenous infusion (Systemic delivery) and intracranial infusion (local delivery) are both CAR T cell delivery strategies that have a significant role in increasing the safety and efficacy of CAR T cell therapy.

Accordingly, specifically targeting the CD19, CD20, CD30, CD33, and BCMA antigens expressing on various B cell lymphomas has reported improved outcomes in the intravenous administration of CAR T cell [150, 151]. In contrast to hematological malignancies, intravenous infusion has demonstrated an inefficient effect on solid tumors. Findings revealed that in solid tumors, intravenous administration has led to CAR T cell accumulation in non-tumoral organs and trafficking disturbance into tumor sites [152, 153]. Undesirably, blood systemic adverse effects have mostly been caused by intravenous infusion of CAR T cells in hematological malignancies or solid tumors.

Encouragingly, successful results of CAR T cell local delivery have been evidenced in solid tumors. For an instance, efficient tumor remission was observed by intracranial infusion of IL13Rα-CAR T cells in glioblastoma [154].

#### CAR T cell production

Appropriate concentration (10^8^–10^9^ cells) of CAR T cell requires impressive efficacy. However, some challenges led to insufficient CAR T cell production. Ex vivo increasing number of CAR T cells is an important phase of CAR T cell manufacturing process after engineering can be improved the function quality of produced cells. For this reason, non-significant expansion of engineered T cells may lead to the insufficient response of CAR T cells.

To expand the CAR T cells, synthetic APCs and stimulatory cytokines like IL-2, IL-7, and IL-15 along with anti-CD3 and anti-CD28 antibodies can be used as expansion trigger factors [155, 156].

Further, patients’ lymphopenia arose from chemotherapy as another major reason for CAR T cell failure production. Importantly, different types of infused T cells impressed the anti-tumor efficacy of CAR T cell therapy. As an instance, central memory T CD8^+^ cells have remarkably indicated the effective cytotoxicity against tumor cells [157–159].

#### CAR T cell persistence and efficacy

Poor persistence or powerless activity of CAR T cell may be related to different causes such as the weak structure of CARs, absence of co-stimulatory molecules, ex vivo and in vivo insufficient expansion or activation, inappropriate infusion strategy, and patients’ lymphopenia. On this ground, some strategies can be used to achieving long persistence and potent anti-tumor activity which are highlighted as two major important features of successful CAR T cell treatment. As described previously, CAR T cells’ persistence, proliferation, and efficacy are promoted from the first generation to the third. Using the third generation of CAR has represented the intense anti-tumor response in comparison to first and second generation [73]. Furthermore, incorporating CD28 or 4-1BB stimulatory domains in CAR structure is a prosperous manner of overcoming poor persistence of CAR T cell and augmenting its functionality [160, 161]. Considerably, the safe and impressive response of CAR T cell, induction of T cell expansion, and high production of IFN-γ have been observed through the use of CD27 co-stimulatory factor [162]. TLR2 is another beneficial co-stimulatory molecule that impressed CAR T cell anti-tumor activity by strengthening the T cell proliferation as well as inflammatory cytokine production [163]. Similarly, the CAR T cells possessing MyD88/CD40 domains have illustrated the potential cytotoxicity against tumor cells [164]. Consequently, CAR T cells’ survival and efficacy can become better by armoring CAR T cells with various powerful co-stimulatory molecules or modifying optimized CAR T cells.

#### Cancer relapse after CAR T cell therapy

Tumor antigen escape, antigen losing, and the short survival of CAR T cells are the possible reasons for cancer relapse; however, principal mechanisms related to relapse are unclear. Despite the successful results of CD19-CAR T cell therapy in various hematological malignancies, cancer relapse has been reported in some patients. Based on this, uncontrollable tumor cell growth, CD19 antigen escape by deletion, and CD19 masking by CAR T cell led to the CD19 inaccessibility which is protected from CAR T cell attack [165–167]. To overcome this challenge, rising frequent responses of the immune system, using other immunotherapy strategies, and promoting CAR T cell expansion and activation can be useful.

#### Autologous and allogeneic CAR T cells

Using the autologous or allogeneic T cells as another principal challenge of CAR T cell therapy has remained unclear. The engineer of autologous CAR T cell is identified as a safe cell therapy with restricted autoimmune responses and GVHD. However, using these types of cells encounters some disadvantages which include high costs and time, elaborate types of equipment, and specialized experts. Disappointingly, the insufficient number of T cells in some patients with lymphopenia and unsuccessful choice for serious disorders are the other challenges of using autologous T cells [168]. Contrarily, allogeneic T cells have more advantages including low-time and low-cost engineering procedures, producing high-quality and high-quantity CAR T cells, and noticeable treatment. Encouragingly, the GVHD effect of using allogeneic T cells can be inhibited through the inactivation of related genes, and also, they can be safely acted after allogeneic HSCT [169].

#### Tumor immune-escape strategies

Tumor cells have various strategies to escape and protect themselves from the immune system which generates different challenges for therapeutic strategies like CAR T cell therapy. As an example, in AML malignancy, AML blasts evade the immune system by recruiting various strategies to include impediment of NK cell cytotoxicity, reduction of tumor antigen expression, enhancement of activated T cell exhaustion, upregulation of anti-apoptotic proteins, upregulation of T cell’s inhibitory ligands like PD-L1, generation of metabolically and immunosuppressive microenvironment, expansion of T-reg cell number, suppression of immune-synapse configuration, and losing the HLA molecules [170–172]. For this reason, various development strategies should be established to overcoming the tumor cells’ immune escape, increasing the effectiveness of CAR T cell therapy by engineering smart generation modified T cells, and improving the CAR T cell therapeutic efficacy in combination with other cancer treatment strategies.

### Side effects

#### Cytokine release syndrome (CRS)

Same as other therapeutic approaches, some side effects also have been reported in CAR T cell therapy. One of the major disadvantages of CAR T cells is related to cytokine production phenomena such as macrophage activation syndrome, hemophagocytic lymphohistiocytosis, and cytokine release syndrome (CRS). CRS may occur in patients with great tumor burden and due to the robust activity of CAR T cells or administration of a high dose of CAR T cell [173]. Strong production of cytokines such as Il-6, IFN-y, IL-1β, GM-CSF, and TNF-α during the CAR T cell function induces the CRS response followed by fevers, hypoxia, myalgia, hypotension, vascular leakage, and neurological disorders [174].

#### On-target/off-tumor toxicity

“On-target/off-tumor” toxicity is another fundamental side effect of CAR T cell that entailed normal tissue devastation which is mediated by targeting antigen-expressing both on tumor cells and healthy cells. CD19 [175], mesothelin [176], Her2 [177], and CAIX [178] are examples of target antigens that led to on-target/off-tumor toxicity through the CAR T cell therapy. Normal B cell damage, so-called B cell aplasia, is the most common example of on-target/off-tumor toxicity caused by anti-CD19 CAR T cell [179]. As well, cardiopulmonary injury due to the destruction of cardiac and pulmonary epithelial is another example that is caused by the anti-Her2 CAR T cells [180].

#### Anaphylaxis

The major population of engineered CAR T cells is composed of the murine ScFv. Based on this, these types of CAR T cells encountering cellular and humoral rejection caused by murine protein immunogenicity which led to the anaphylaxis outbreak. Acute anaphylaxis as an immediate interaction occurs because of the host immune system reaction after recognizing the foreign component has entered [181].

#### Neurotoxicity

Neurotoxicity is nervous system toxicity accompanied by expressive aphasia, delirium, seizure, and confusion symptoms. The incidence etiology of neurotoxicity is unclear; however, it may happen by inflammation derived from cytokine overproduction which has been mostly reported in patients treated by the CD19-CAR T cell [182].

#### Insertional oncogenesis

Besides, insertional oncogenesis is another side effect that comes about through the transferring of CAR transgene into T cells by lentiviral or retroviral vectors which can increase the induced malignant transformation risk. Fortunately, the incidence rate of insertional oncogenesis is less; however, considering accurate and safe measures is required in the CAR T cell engineering procedure [183].

#### Hematological toxicity

Hematological toxicity as another adverse effect of CAR T cell therapy is caused after allogeneic hematopoietic stem cell transplantation (alloHSCT) and CAR T cell administration. Neutropenia, anemia, and thrombocytopenia are the reported examples of hematopoietic system toxicities after CAR T cell therapy. Normal HSPC destruction by CAR T cell due to the presence of common antigen on malignant cells and HSPCs like CD33 is identified as hematological toxicity in AML patients [184, 185].

### CAR T cell therapy challenges regarding their toxicity

#### CAR T cell toxicity management

To mitigate the toxicity and improve the clinical application and efficacy of CAR T cell therapy, various approaches have been developed. Some strategies are used to control the CAR T cell activity with switch-off mechanisms to avoid the CAR T cell overactivity, and some of them have been developed to decrease mentioned side effects derived from CAR T cells. In addition, using the high-affinity ScFv, humanized or human-derived ScFv rather than murine, utilization of local delivery instead of intravenously, and optimizing the CAR T cell manufacturing and infusion process can be improved the clinical therapeutic applications of CAR T cell [182, 186].

#### Inhibition of CRS and Neurotoxicity

To inhibit the incidence of CRS, anti-inflammatory corticosteroid drugs like dexamethasone as a first choice and cytokine blockade antibodies can be used. Tocilizumab or sarilumab as an anti-IL-6 receptor antibody can be administered to suppressing the effects of IL-6 production [187, 188]. Moreover, ibrutinib has a role in reducing inflammatory cytokine production like IFN-γ [189]. Anakinra as an IL-1 receptor pharmacological antagonist and lenzilumab as an anti-GM-CSF blockade antibody reduce the CRS and neurotoxicity by neutralizing these cytokines [190, 191].

#### Inhibition of anaphylaxis

Intending to overcome anaphylaxis toxicity, efforts for using the humanized or human ScFv are undergoing, to improve the survival and function of CAR T cells [192].

#### Inhibition of hematological toxicity

In hematological malignancies, the incidence of hematological toxicity is possible due to the CAR T cell activity. To overcome this toxicity, common antigens between leukemic cells and HSPCs should be removed from HSPCs by gene-editing methods like CRISPR/Cas9 system and then used for alloHSCT. As an example, in a CD33^+^ AML patient, alloHSCT with CD33^negative^ HSPCs along with anti-CD33-CAR T cell therapy can eradicate the CD33^+^ blasts without effect on CD33^neagative^ HSPCs which results in reducing the hematopoietic toxicity [114, 193]. Collectively, combinational therapy of alloHSCT and CAR T cell therapy as a novel therapeutic strategy would be improved cancer treatment like AML.

#### Inhibition of “on-target/off-tumor” toxicity and CAR T cell overactivity

Various controllable strategies have been developed to switch off the CAR T cell, optimize the CAR T cell function, and prevent the CAR T cell overactivation which mitigated the CAR T cell-derived side effects like on-target/off-tumor toxicity. Accordingly, development strategies include the mRNA electroporation delivery system, suicide genes, CRISPR/Cas9 gene-editing strategy, and smart or multi-targeted CAR T cells (“Advanced generations of CAR T cell” section).

##### mRNA electroporation

CAR T cell transient delivery systems like mRNA electroporation strategy as well as transposon/transposase are used to limit CAR T cell expression and activation. A biodegradable CAR like RNA-CAR123 is an example of using an mRNA electroporation mechanism that gradually decays the CAR T cell expression and activation in AML cases [193].

##### Suicide genes

Employing the suicide genes allows decaying the CAR T cell activity selectively which may be critical to prevent the toxicity. The herpes simplex virus thymidine kinase was the first validated suicide gene in human trial studies which elicits the CAR T cell destruction by catalyzing the ganciclovir phosphorylation as an acyclic nucleoside analog. However, immunogenicity caused by herpes simplex virus thymidine kinase is identified as a limitation of this strategy [194]. Fas and caspase 9 (ICasp9) termed as death molecules are the dimerize suicide genes used to depleting CAR T cells selectively. A small-dimerizing molecule like AP1903 causes the Fas or caspase 9 dimerization followed by downstream caspase activation and the apoptotic stimulation in modified T cells [195, 196].

##### Eliminating genes

Eliminating genes are known as another mechanism of CAR T cell-selective exhaustion. To induce the CAR T cell death, CD20 or EGFR identified as cell surface antigens can be used in CAR structure. Then, the infusion of CD20 (rituximab) or EGFR (cetuximab) mAbs trigger the CAR T cell death through their interaction with mentioned eliminating genes [197, 198].

##### CRISPR/Cas9 genome editing system

An important gene-modifying strategy with a high potential operation is Clustered Regularly Interspaced Short Palindromic Repeats CRISPR/CRISPR-Associated 9 (Cas9). This strategy is used to developing the next-generation CAR T cells with high efficacy and safety by genome manipulation of antigens, enzymes, checkpoints, and cytokines [61, 64].

## Advanced generations of CAR T cell

Nowadays, to improve the CAR T cell efficacy and safety, next-generation CAR T cells have been developed so-called smart, programmable, or multi-targeted CAR T cells. These smart CARs are including Tandem CAR, Dual CAR, Universal CAR, SUPRA CAR, SynNotch CAR, TRUCKs, Split CAR, physiological CAR, and iCAR which have various roles in targeting multiple antigens on tumor cells, controlling CAR T cell function, and switching off CARs [58, 61, 199]. As a suggestion, smart CARs possessing the mentioned capabilities can be employed in AML CAR T cell therapy and decrease the related side effects as described above. Concerning variously identified target antigens on AML blasts, multi-targeted CARs like Tandem, dual, SUPRA, or universal CARs can be used to targeting two or more antigens concurrently which co-expressed on tumor cells without on-target/off-tumor toxicity on normal tissues [200].

In a study by Cartellieri et al. [201], a flexible CAR platform so-called universal CAR T cell was engineered to target CD33 and/or CD123 AML blasts in vitro and in vivo. Universal CAR regulating the CAR T cell function by on/off switching mechanism. As a result, universal CAR T cell lyses the AML blasts and AML cell lines potentially by dual-targeting the CD33 and CD123 antigens. Interestingly, no on-target/off-tumor toxicity and xenogeneic graft-versus-host disease were reported in using the universal CAR. In practice, two or multiple antigens targeted simultaneously can be reduced the risk of tumor escape, on-target/off-tumor toxicity, and increased the specificity of antigen targeting.

Considering the lack of proper specific targets of AML, new potential targets can be discovered to improve the AML treatment. Hence, in a study by Perna et al. [200], the discovery and analysis of AML new target antigens were conducted by combining the proteomic and genomic resulted from data from AML patients and healthy volunteers to finding the ideal targets of AML for CAR T cell therapy. As a consequence, four hopeful and potential target antigens were found to include ADGRE2, CCR1, CD70, and LILRB2 which could be multiple targeted by engineering appropriate smart CARs. More than that, they predicted identified antigen overexpression on the majority of AML blasts along with limited expression on normal cells and activated T cells, expression in a majority of AML patients, and less off-tumor toxicity on healthy cells. Consequently, recognizing the new target antigens in AML and establishing the powerful smart CAR T cells can hopefully increase the success rate of CAR T cell therapy in AML patients.

## Concluding remarks

In the last decades, the importance of cancer treatment has led to developing immunotherapy-based approaches [7]. T cell equipped with CAR named CAR T cell is one of the potential adoptive cell therapy evolved for solid tumors and hematological malignancies and indicated the hopeful remission [11]. Interestingly, the hopeful effectiveness of CAR T cells has been reported from several preclinical and trial studies. Various CAR T cells have been engineered for different targets of malignancies in which the FDA-approved CD19-CAR T cells as a common and effective type emphasizes the utilization of CAR T cell therapy. Lastly, multiple CAR T cells have been designed for diverse extracellular and intracellular antigens of AML with encouraging results such as potent cytotoxicity, high persistence, and proliferation, increased cytokine production, less toxicity on normal cells, and prevention of tumor immune escape. Nonetheless, the same as other cancer treatment approaches, there are some challenges and toxicity related to the CAR T cell which can be overcome by CARs modifying or armoring strategies. In AML, to overcome the tumor immune-escape challenge and improve the CAR T cell functionality, some strategies can be provided such as avoiding the T-reg cell expansion, upregulating the proapoptotic proteins, increasing the tumor antigens expression, and blocking the inhibitory checkpoints. Moreover, designing the CAR T cell through high-quality and accurate procedures would improve the proliferation, persistence, and performance of CAR T cells to overcome the related challenges. Also, to prevent the common side effects of CAR T cell which include CRS, on-target/off-tumor toxicity, anaphylaxis, neurotoxicity, insertional oncogenesis, and hematological toxicity, some efficient strategies need to be discovered. Based on this, CRISPR/Cas9 known as a potent gene-editing strategy was used in alloHSCT and CAR T cell therapy to decrease the hematological and on-target/off-tumor toxicity by knocking out the related genes [193]. Additionally, the switching-off mechanisms like mRNA electroporation, suicide, or eliminating genes as a potent controllable mechanism of CAR T cell overactivity and programmable or multi-targeted CAR T cells as powerful armoring CAR T cells have been developed to enhance the safety, efficacy, and cytotoxic specificity [58, 61].

In conclusion, since there is no approved CAR T cell therapy approach for AML, discovering the predisposed and potential AML target antigens and performing the various strategies to improving the CAR T cells’ safety and efficacy can lead to a breakthrough in AML CAR T cell therapy.

## Data Availability

Not applicable.

## Abbreviations

CAR: Chimeric antigen receptor
GvHD: Graft-versus-host disease
CAR-NK: CAR-transduced NK cells
NK cells: Natural killer cells
ICIs: Immune checkpoint inhibitors
ACTs: Adaptive cell therapies
NHL: Non-Hodgkin lymphomas
DLBCL: Diffused large B cell lymphoma
FL: Follicular lymphoma
ALL: Lymphoblastic leukemia
MM: Multiple myeloma
AML: Acute myeloid leukemia
CRS: Cytokine release syndrome
CTLs: Cytotoxic T lymphocytes
NCRs: Cytotoxicity receptors
SHP-1: SH-2 containing protein tyrosine phosphatase
ITIMs: Immunoreceptor tyrosine-based inhibitory motifs
hESCs: Human embryonic stem cells
SCGM: Stem Cell Growth Medium
bNHL: B cell non-Hodgkin’s lymphoma
RCC: Renal cell carcinoma
CAIX: Carbonic anhydrase IX
EpCAM: Epithelial cell adhesion molecule
iCAS9: Inducible caspase 9

## References

[1] Hassan G, Seno M (2020). Blood and cancer: cancer stem cells as origin of hematopoietic cells in solid tumor microenvironments. Cells.

[2] Cornelissen JJ, Blaise D (2016). Hematopoietic stem cell transplantation for patients with AML in first complete remission. Blood J Am Soc Hematol.

[3] Mo JS, Park HW, Guan KL (2014). The Hippo signaling pathway in stem cell biology and cancer. EMBO Rep.

[4] Pui C-H, Carroll WL, Meshinchi S, Arceci RJ (2011). Biology, risk stratification, and therapy of pediatric acute leukemias: an update. J Clin Oncol.

[5] Shomali N, Gharibi T, Vahedi G, Mohammed RN, Mohammadi H, Salimifard S, Marofi F (2020). Mesenchymal stem cells as carrier of the therapeutic agent in the gene therapy of blood disorders. J Cell Physiol.

[6] Sambi M, Bagheri L, Szewczuk MR. Current challenges in cancer immunotherapy: multimodal approaches to improve efficacy and patient response rates. J Oncol. 2019;2019:4508794. 10.1155/2019/4508794 PMC6420990 30941175

[7] Winer ES, Stone RM (2019). Novel therapy in acute myeloid leukemia (AML): moving toward targeted approaches. Ther Adv Hematol.

[8] Larson RA, Sievers EL, Stadtmauer EA, Löwenberg B, Estey EH, Dombret H, Theobald M, Voliotis D, Bennett JM, Richie M (2005). Final report of the efficacy and safety of gemtuzumab ozogamicin (Mylotarg) in patients with CD33-positive acute myeloid leukemia in first recurrence. Cancer.

[9] Beyar-Katz O, Gill S (2018). Novel approaches to acute myeloid leukemia immunotherapy. Clin Cancer Res.

[10] Hofmann S, Schubert M-L, Wang L, He B, Neuber B, Dreger P, Müller-Tidow C, Schmitt M (2019). Chimeric antigen receptor (CAR) T cell therapy in acute myeloid leukemia (AML). J Clin Med.

[11] Gross G, Waks T, Eshhar Z (1989). Expression of immunoglobulin-T-cell receptor chimeric molecules as functional receptors with antibody-type specificity. Proc Natl Acad Sci.

[12] Au R (2017). Immunooncology: can the right chimeric antigen receptors T-cell design be made to cure all types of cancers and will it be covered?. J Pharm.

[13] Vasekar M, Rizvi S, Liu X, Vrana KE, Zheng H (2016). Novel immunotherapies for hematological malignancies. Curr Mol Pharmacol.

[14] June CH, Sadelain M (2018). Chimeric antigen receptor therapy. N Engl J Med.

[15] Lancet JE, Cortes JE, Hogge DE, Tallman MS, Kovacsovics TJ, Damon LE, Komrokji R, Solomon SR, Kolitz JE, Cooper M (2014). Phase 2 trial of CPX-351, a fixed 5: 1 molar ratio of cytarabine/daunorubicin, vs cytarabine/daunorubicin in older adults with untreated AML. Blood.

[16] Lancet JE, Uy GL, Cortes JE, Newell LF, Lin TL, Ritchie EK, Stuart RK, Strickland SA, Hogge D, Solomon SR (2018). CPX-351 (cytarabine and daunorubicin) liposome for injection versus conventional cytarabine plus daunorubicin in older patients with newly diagnosed secondary acute myeloid leukemia. J Clin Oncol.

[17] Shaffer BC, Gillet J-P, Patel C, Baer MR, Bates SE, Gottesman MM (2012). Drug resistance: still a daunting challenge to the successful treatment of AML. Drug Resist Updates.

[18] Munchhof MJ, Li Q, Shavnya A, Borzillo GV, Boyden TL, Jones CS, LaGreca SD, Martinez-Alsina L, Patel N, Pelletier K (2011). Discovery of PF-04449913, a potent and orally bioavailable inhibitor of smoothened. ACS Med Chem Lett.

[19] Queiroz K, Ruela-de-Sousa R, Fuhler G, Aberson H, Ferreira C, Peppelenbosch M, Spek C (2010). Hedgehog signaling maintains chemoresistance in myeloid leukemic cells. Oncogene.

[20] Sadarangani A, Pineda G, Lennon KM, Chun H-J, Shih A, Schairer AE, Goff DJ, Prashad SL, Geron I, Wall R (2015). GLI2 inhibition abrogates human leukemia stem cell dormancy. J Transl Med.

[21] Martinelli G, Oehler VG, Papayannidis C, Courtney R, Shaik MN, Zhang X, O'Connell A, McLachlan KR, Zheng X, Radich J (2015). Treatment with PF-04449913, an oral smoothened antagonist, in patients with myeloid malignancies: a phase 1 safety and pharmacokinetics study. Lancet Haematol.

[22] Stone RM (2018). What FLT3 inhibitor holds the greatest promise?. Best Pract Res Clin Haematol.

[23] Stone RM, Mandrekar SJ, Sanford BL, Laumann K, Geyer S, Bloomfield CD, Thiede C, Prior TW, Döhner K, Marcucci G (2017). Midostaurin plus chemotherapy for acute myeloid leukemia with a FLT3 mutation. N Engl J Med.

[24] Konopleva M, Letai A (2018). BCL-2 inhibition in AML: an unexpected bonus?. Blood.

[25] Ragon BK, DiNardo CD (2017). Targeting IDH1 and IDH2 mutations in acute myeloid leukemia. Curr Hematol Malig Rep.

[26] Konopleva M, Pollyea DA, Potluri J, Chyla B, Hogdal L, Busman T, McKeegan E, Salem AH, Zhu M, Ricker JL (2016). Efficacy and biological correlates of response in a phase II study of venetoclax monotherapy in patients with acute myelogenous leukemia. Cancer Discov.

[27] Bykov VJ, Zhang Q, Zhang M, Ceder S, Abrahmsen L, Wiman KG (2016). Targeting of mutant p53 and the cellular redox balance by APR-246 as a strategy for efficient cancer therapy. Front Oncol.

[28] Döhner H, Lübbert M, Fiedler W, Fouillard L, Haaland A, Brandwein JM, Lepretre S, Reman O, Turlure P, Ottmann OG (2014). Randomized, phase 2 trial of low-dose cytarabine with or without volasertib in AML patients not suitable for induction therapy. Blood.

[29] Garcia-Manero G, Fenaux P, Al-Kali A, Baer MR, Sekeres MA, Roboz GJ, Gaidano G, Scott BL, Greenberg P, Platzbecker U (2016). Rigosertib versus best supportive care for patients with high-risk myelodysplastic syndromes after failure of hypomethylating drugs (ONTIME): a randomised, controlled, phase 3 trial. Lancet Oncol.

[30] David L, Fernandez-Vidal A, Bertoli S, Grgurevic S, Lepage B, Deshaies D, Prade N, Cartel M, Larrue C, Sarry J-E (2016). CHK1 as a therapeutic target to bypass chemoresistance in AML. Sci Signal.

[31] Uras IZ, Walter GJ, Scheicher R, Bellutti F, Prchal-Murphy M, Tigan AS, Valent P, Heidel FH, Kubicek S, Scholl C (2016). Palbociclib treatment of FLT3-ITD+ AML cells uncovers a kinase-dependent transcriptional regulation of FLT3 and PIM1 by CDK6. Blood.

[32] Maslak PG, Dao T, Bernal Y, Chanel SM, Zhang R, Frattini M, Rosenblat T, Jurcic JG, Brentjens RJ, Arcila ME (2018). Phase 2 trial of a multivalent WT1 peptide vaccine (galinpepimut-S) in acute myeloid leukemia. Blood Adv.

[33] Yamaguchi M, Takezako N, Kiguchi T, Miyawaki S, Heike Y, Mitsuki K, Yoshida T, Liew EL, Naoe T (2018). Phase II Trial of a Peptide Vaccine, Ocv-501 in Elderly Patients with Acute Myeloid Leukemia. Am Soc Hematology.

[34] van de Loosdrecht AA, van Wetering S, Santegoets SJ, Singh SK, Eeltink CM, den Hartog Y, Koppes M, Kaspers J, Ossenkoppele GJ, Kruisbeek AM (2018). A novel allogeneic off-the-shelf dendritic cell vaccine for post-remission treatment of elderly patients with acute myeloid leukemia. Cancer Immunol Immunother.

[35] Anguille S, Van de Velde AL, Smits EL, Van Tendeloo VF, Juliusson G, Cools N, Nijs G, Stein B, Lion E, Van Driessche A (2017). Dendritic cell vaccination as postremission treatment to prevent or delay relapse in acute myeloid leukemia. Blood.

[36] Rosenblatt J, Stone RM, Uhl L, Neuberg D, Joyce R, Levine JD, Arnason J, McMasters M, Luptakova K, Jain S (2016). Individualized vaccination of AML patients in remission is associated with induction of antileukemia immunity and prolonged remissions. Sci Transl Med.

[37] Sievers EL, Larson RA, Stadtmauer EA, Estey E, Löwenberg B, Dombret H, Karanes C, Theobald M, Bennett JM, Sherman ML (2001). Efficacy and safety of gemtuzumab ozogamicin in patients with CD33-positive acute myeloid leukemia in first relapse. J Clin Oncol.

[38] Medeiros BC, Tanaka TN, Balaian L, Bashey A, Guzdar A, Li H, Messer K, Ball ED (2018). A phase I/II trial of the combination of azacitidine and gemtuzumab ozogamicin for treatment of relapsed acute myeloid leukemia. Clin Lymphoma myeloma Leuk.

[39] E.M. Stein, R.B. Walter, H.P. Erba, A.T. Fathi, A.S. Advani, J.E. Lancet, F. Ravandi, T. Kovacsovics, D.J. DeAngelo, D. Bixby, S. Faderl, A.P. Jillella, P.A. Ho, A phase 1 trial of vadastuximab talirine as monotherapy in patients with CD33-positive acute myeloid leukemia. Blood, The Journal of the American Society of Hematology 131 (2018) 387-396. 10.1182/blood-2017-06-789800 PMC5813721 29196412

[40] He SZ, Busfield S, Ritchie DS, Hertzberg MS, Durrant S, Lewis ID, Marlton P, McLachlan AJ, Kerridge I, Bradstock KF, Kennedy G, Boyd AW, Yeadon TM, Lopez AF, Ramshaw HS, Iland H, Bamford S, Barnden M, DeWitte M, Basser R, Roberts AW (2015). A Phase 1 study of the safety, pharmacokinetics and anti-leukemic activity of the anti-CD123 monoclonal antibody CSL360 in relapsed, refractory or high-risk acute myeloid leukemia. Leuk Lymphoma.

[41] Kovtun Y, Jones GE, Adams S, Harvey L, Audette CA, Wilhelm A, Bai C, Rui L, Laleau R, Liu F, Ab O, Setiady Y, Yoder NC, Goldmacher VS, Chari RVJ, Pinkas J, Chittenden T (2018). A CD123-targeting antibody-drug conjugate, IMGN632, designed to eradicate AML while sparing normal bone marrow cells. Blood Adv.

[42] Li F, Sutherland MK, Yu C, Walter RB, Westendorf L, Valliere-Douglass J, Pan L, Cronkite A, Sussman D, Klussman K, Ulrich M, Anderson ME, Stone IJ, Zeng W, Jonas M, Lewis TS, Goswami M, Wang SA, Senter PD, Law CL, Feldman EJ, Benjamin DR (2018). Characterization of SGN-CD123A, A potent CD123-directed antibody-drug conjugate for acute myeloid leukemia. Mol Cancer Ther.

[43] Harrington KH, Gudgeon CJ, Laszlo GS, Newhall KJ, Sinclair AM, Frankel SR, Kischel R, Chen G, Walter RB (2015). The broad anti-aml activity of the CD33/CD3 BiTE antibody construct, AMG 330, is impacted by disease stage and risk. PLoS One.

[44] Uy GL, Rettig MP, Vey N, Godwin J, Foster MC, Rizzieri DA, Arellano ML, Topp MS, Huls G, Jongen-Lavrencic M, Martinelli G, Paolini S, Ciceri F, Carrabba MG, Sweet KL, Ravandi F, Church SE, Vadakekolathu J, Rutella S, Sun J, Yang K, Baughman J, Curtis T, Timmeny E, Cali K, Tran K, Muth J, La Motte-Mohs R, Poirot C, Pallis A, Cesano A, Bonvini E, Wigginton J, Lowenberg B, Davidson-Moncada JK, DiPersio JF (2018). Phase 1 cohort expansion of flotetuzumab, a CD123 × CD3 bispecific Dart® protein in patients with relapsed/refractory acute myeloid leukemia (AML). Blood.

[45] Zeidan AM, Knaus HA, Robinson TM, Towlerton AMH, Warren EH, Zeidner JF, et al. A multi-center phase I trial of ipilimumab in patients with myelodysplastic syndromes following hypomethylating agent failure. Clinical Cancer Research. 2018;24:3519–27. 10.1158/1078-0432.CCR-17-3763 PMC6680246 29716921

[46] B.SN. Daver, Garcia-Manero G, Cortes JE, Ravandi F, Jabbour EJ, Hendrickson S, Pierce S, Ning J, Konopleva M, Andreeff M. Phase IB/II study of nivolumab in combination with azacytidine (AZA) in patients (pts) with relapsed acute myeloid leukemia (AML). 2016.

[47] G.-M.G, Daver NG, Basu S, Cortes JE, Ravandi F, Kadia TM, Konopleva MY, Jabbour EJ, DiNardo CD, Assi R, Pierce SA, Safety, efficacy, and biomarkers of response to azacitidine (AZA) with nivolumab (Nivo) and AZA with nivo and ipilimumab (Ipi) in relapsed/refractory acute myeloid leukemia: a non-randomized, phase 2 study. 2018.

[48] Naserian S, Leclerc M, Shamdani S, Uzan G (2020). Current preventions and treatments of aGVHD: from pharmacological prophylaxis to innovative therapies. Front Immunol.

[49] Stahl M, Goldberg AD (2019). Immune checkpoint inhibitors in acute myeloid leukemia: novel combinations and therapeutic targets. Curr Oncol Rep.

[50] Narayan R, Olsson N, Wagar LE, Medeiros BC, Meyer E, Czerwinski D, Khodadoust MS, Zhang L, Schultz L, Davis MM, Elias JE, Levy R (2019). Acute myeloid leukemia immunopeptidome reveals HLA presentation of mutated nucleophosmin. PLoS One.

[51] Alejandro Madrigal J, Barber LD (2016). Matching inside and outside the HLA molecule in allogeneic hematopoietic stem cell transplantation. Haematologica.

[52] Gupta V, Tallman MS, Weisdorf DJ (2011). Allogeneic hematopoietic cell transplantation for adults with acute myeloid leukemia: myths, controversies, and unknowns. Blood.

[53] Kassim AA, Savani BN (2017). Hematopoietic stem cell transplantation for acute myeloid leukemia: a review. Hematol Oncol Stem Cell Ther.

[54] Zhu CY, Chen GF, Zhou W, Hou C, Wang XK, Wang FY, Yang N, Wang L, Fang S, Luo L, Guan LX, Zhang R, Liu YC, Dou LP, Gao CJ (2019). Outcome and prognostic factors of high-risk acute myeloid leukemia after allogeneic hematopoietic stem cell transplantation. Ann Transplantat.

[55] Sweeney C, Vyas P (2019). The Graft-Versus-Leukemia Effect in AML. Front Oncol.

[56] Lipof JJ, Loh KP, O'Dwyer K, Liesveld JL (2018). Allogeneic hematopoietic cell transplantation for older adults with acute myeloid leukemia. Cancers (Basel).

[57] Li D, Li X, Zhou W-L, Huang Y, Liang X, Jiang L, Yang X, Sun J, Li Z, Han W-D, Wang W (2019). Genetically engineered T cells for cancer immunotherapy. Signal Transduct Target Ther.

[58] Tahmasebi S, Elahi R, Esmaeilzadeh A (2019). Solid tumors challenges and new insights of CAR T cell engineering. Stem Cell Rev Rep.

[59] Marofi F, Tahmasebi S, Rahman HS, Kaigorodov D, Markov A, Yumashev AV, Shomali N, Chartrand MS, Pathak Y, Mohammed RN, Jarahian M, Motavalli R, Motavalli Khiavi F (2021). Any closer to successful therapy of multiple myeloma? CAR-T cell is a good reason for optimism. Stem Cell Res Ther.

[60] Marofi F, Rahman HS, Thangavelu L, Dorofeev A, Bayas-Morejón F, Shirafkan N, Shomali N, Chartrand MS, Jarahian M, Vahedi G (2021). Renaissance of armored immune effector cells, CAR-NK cells, brings the higher hope for successful cancer therapy. Stem Cell Res Ther.

[61] Elahi R, Khosh E, Tahmasebi S, Esmaeilzadeh A (2018). Immune cell hacking: challenges and clinical approaches to create smarter generations of chimeric antigen receptor T cells. Front Immunol.

[62] Srivastava S, Riddell SR (2015). Engineering CAR-T cells: design concepts. Trends Immunol.

[63] Hock RA, Miller AD (1986). Retrovirus-mediated transfer and expression of drug resistance genes in human haematopoietic progenitor cells. Nature.

[64] Eyquem J, Mansilla-Soto J, Giavridis T, van der Stegen SJ, Hamieh M, Cunanan KM, Odak A, Gonen M, Sadelain M (2017). Targeting a CAR to the TRAC locus with CRISPR/Cas9 enhances tumour rejection. Nature.

[65] Toneguzzo F, Keating A (1986). Stable expression of selectable genes introduced into human hematopoietic stem cells by electric field-mediated DNA transfer. Proc Natl Acad Sci U S A.

[66] Schaefer-Ridder M, Wang Y, Hofschneider PH (1982). Liposomes as gene carriers: efficient transformation of mouse L cells by thymidine kinase gene. Science (New York, N.Y.).

[67] Chmielewski M, Hombach AA, Abken H (2014). Of CARs and TRUCKs: chimeric antigen receptor (CAR) T cells engineered with an inducible cytokine to modulate the tumor stroma. Immunol Rev.

[68] Koneru M, O'Cearbhaill R, Pendharkar S, Spriggs DR, Brentjens RJ (2015). A phase I clinical trial of adoptive T cell therapy using IL-12 secreting MUC-16(ecto) directed chimeric antigen receptors for recurrent ovarian cancer. J Transl Med.

[69] Zhang E, Xu H (2017). A new insight in chimeric antigen receptor-engineered T cells for cancer immunotherapy. J Hematol Oncol.

[70] Marofi F, Motavalli R, Safonov VA, Thangavelu L, Yumashev AV, Alexander M, Shomali N, Chartrand MS, Pathak Y, Jarahian M (2021). CAR T cells in solid tumors: challenges and opportunities. Stem Cell Res Ther.

[71] Grupp SA, Kalos M, Barrett D, Aplenc R, Porter DL, Rheingold SR, Teachey DT, Chew A, Hauck B, Wright JF, Milone MC, Levine BL, June CH (2013). Chimeric antigen receptor–modified t cells for acute lymphoid leukemia. N Engl J Med.

[72] Kochenderfer JN, Wilson WH, Janik JE, Dudley ME, Stetler-Stevenson M, Feldman SA, Maric I, Raffeld M, Nathan DA, Lanier BJ, Morgan RA, Rosenberg SA (2010). Eradication of B-lineage cells and regression of lymphoma in a patient treated with autologous T cells genetically engineered to recognize CD19. Blood.

[73] Porter DL, Levine BL, Kalos M, Bagg A, June CH (2011). Chimeric antigen receptor–modified T cells in chronic lymphoid leukemia. N Engl J Med.

[74] Bishop MR, Maziarz RT, Waller EK. Tisagenlecleucel in relapsed/refractory diffuse large B-cell lymphoma patients without measurable disease at infusion. Blood advances. 2019;3:2230–6. 10.1182/bloodadvances.2019000151 PMC6650727 31332046

[75] Maus MV, Levine BL (2016). Chimeric Antigen Receptor T-Cell Therapy for the Community Oncologist. Oncologist.

[76] Schuster SJ, Svoboda J, Nasta S, Porter DL, Mato A, Shah GD, Landsburg DJ, Chong EA, Lacey SF, Melenhorst JJ, Chew A, Hasskarl J, Shah NN, Wasik MA, Marcucci K, Zheng Z, Levine B, June CH (2015). Phase IIa trial of chimeric antigen receptor modified T cells directed against CD19 (CTL019) in patients with relapsed or refractory CD19+ lymphomas. J Clin Oncol.

[77] Fry TJ, Shah NN, Orentas RJ, Stetler-Stevenson M, Yuan CM, Ramakrishna S, et al. CD22-targeted CAR T cells induce remission in B-ALL that is naive or resistant to CD19-targeted CAR immunotherapy. Nature medicine. 2018;24:20–8. 10.1038/nm.4441 PMC5774642 29155426

[78] Till BG, Jensen MC, Wang J, Chen EY, Wood BL, Greisman HA, Qian X, James SE, Raubitschek A, Forman SJ, Gopal AK, Pagel JM, Lindgren CG, Greenberg PD, Riddell SR, Press OW (2008). Adoptive immunotherapy for indolent non-Hodgkin lymphoma and mantle cell lymphoma using genetically modified autologous CD20-specific T cells. Blood.

[79] Zhang WY, Guo YL, Dai HR, Yang QM, Zhang YJ, Zhang Y, Chen MX, Wang CM, Feng KC, Li SX, W.Y (2016). Treatment of CD20-directed chimeric antigen receptor-modified T cells in patients with relapsed or refractory B-cell non-Hodgkin lymphoma: an early phase IIa trial report. Signal Transduct Targeted Ther.

[80] Jain N, O'Brien S (2016). Targeted therapies for CLL: practical issues with the changing treatment paradigm. Blood Rev.

[81] Kochenderfer JN, Dudley ME, Kassim SH, Somerville RP, Carpenter RO, Stetler-Stevenson M, Yang JC, Phan GQ, Hughes MS, Sherry RM, Raffeld M, Feldman S, Lu L, Li YF, Ngo LT, Goy A, Feldman T, Spaner DE, Wang ML, Chen CC, Kranick SM, Nath A, Nathan DA, Morton KE, Toomey MA, Rosenberg SA (2015). Chemotherapy-refractory diffuse large B-cell lymphoma and indolent B-cell malignancies can be effectively treated with autologous T cells expressing an anti-CD19 chimeric antigen receptor. J Clin Oncol.

[82] Ramos CA, Heslop HE, Brenner MK (2016). CAR-T cell therapy for lymphoma. Annu Rev Med.

[83] Ali SA, Shi V, Maric I, Wang M, Stroncek DF, Rose JJ, Brudno JN, Stetler-Stevenson M, Feldman SA, Hansen BG (2016). T cells expressing an anti–B-cell maturation antigen chimeric antigen receptor cause remissions of multiple myeloma. Blood.

[84] Guo B, Chen M, Han Q, Hui F, Dai H, Zhang W, Zhang Y, Wang Y, Zhu H, Han W (2016). CD138-directed adoptive immunotherapy of chimeric antigen receptor (CAR)-modified T cells for multiple myeloma. J Cell Immunother.

[85] Gomes-Silva D, Atilla E, Atilla PA, Mo F, Tashiro H, Srinivasan M, Lulla P, Rouce RH, Cabral JM, Ramos CA (2019). CD7 CAR T cells for the therapy of acute myeloid leukemia. Mol Ther.

[86] Kenderian SS, Ruella M, Shestova O, Klichinsky M, Aikawa V, Morrissette JJ, Scholler J, Song D, Porter DL, Carroll M (2015). CD33-specific chimeric antigen receptor T cells exhibit potent preclinical activity against human acute myeloid leukemia. Leukemia.

[87] O'Hear C, Heiber JF, Schubert I, Fey G, Geiger TL (2015). Anti-CD33 chimeric antigen receptor targeting of acute myeloid leukemia. Haematologica.

[88] Yoshida T, Mihara K, Takei Y, Yanagihara K, Kubo T, Bhattacharyya J, Imai C, Mino T, Takihara Y, Ichinohe T (2016). All-trans retinoic acid enhances cytotoxic effect of T cells with an anti-CD38 chimeric antigen receptor in acute myeloid leukemia. Clin Transl Immunol.

[89] Drent E, Groen RW, Noort WA, Themeli M, Lammerts van Bueren JJ, Parren PW, Kuball J, Sebestyen Z, Yuan H, de Bruijn J, van de Donk NW, Martens AC, Lokhorst HM, Mutis T (2016). Pre-clinical evaluation of CD38 chimeric antigen receptor engineered T cells for the treatment of multiple myeloma. Haematologica.

[90] Casucci M, di Robilant BN, Falcone L, Camisa B, Norelli M, Genovese P, Gentner B, Gullotta F, Ponzoni M, Bernardi M (2013). CD44v6-targeted T cells mediate potent antitumor effects against acute myeloid leukemia and multiple myeloma. Blood.

[91] Nolte MA, Van Olffen RW, Van Gisbergen KP, Van Lier RA (2009). Timing and tuning of CD27–CD70 interactions: the impact of signal strength in setting the balance between adaptive responses and immunopathology. Immunol Rev.

[92] Sauer T, Parikh K, Sharma S, Omer B, Gottschalk S, Rooney CM (2019). CD70-specific CAR T cells have potent activity against acute myeloid leukemia (AML) without HSC toxicity. American Society of Hematology Washington, DC.

[93] Pizzitola I, Anjos-Afonso F, Rouault-Pierre K, Lassailly F, Tettamanti S, Spinelli O, Biondi A, Biagi E, Bonnet D (2014). Chimeric antigen receptors against CD33/CD123 antigens efficiently target primary acute myeloid leukemia cells in vivo. Leukemia.

[94] Gill S, Tasian SK, Ruella M, Shestova O, Li Y, Porter DL, Carroll M, Danet-Desnoyers G, Scholler J, Grupp SA (2014). Preclinical targeting of human acute myeloid leukemia and myeloablation using chimeric antigen receptor–modified T cells. Blood.

[95] Chien CD, Sauter CT, Ishii K, Nguyen SM, Shen F, Tasian SK, Chen W, Dimitrov DS, Fry TJ (2016). Preclinical development of FLT3-redirected chimeric antigen receptor T cell immunotherapy for acute myeloid leukemia. Am Soc Hematology.

[96] Jetani H, Garcia-Cadenas I, Nerreter T. CAR T-cells targeting FLT3 have potent activity against FLT3(-)ITD(+) AML and act synergistically with the FLT3-inhibitor crenolanib. 2018;32:1168–79. 10.1038/s41375-018-0009-0 29472720

[97] Wang J, Chen S, Xiao W, Li W, Wang L, Yang S, Wang W, Xu L, Liao S, Liu W (2018). CAR-T cells targeting CLL-1 as an approach to treat acute myeloid leukemia. J Hematol Oncol.

[98] Laborda E, Mazagova M, Shao S, Wang X, Quirino H, Woods A, Hampton E, Rodgers D, Kim C, Schultz P (2017). Development of a chimeric antigen receptor targeting c-type lectin-like molecule-1 for human acute myeloid leukemia. Int J Mol Sci.

[99] Peinert S, Prince H, Guru P, Kershaw M, Smyth M, Trapani J, Gambell P, Harrison S, Scott A, Smyth F (2010). Gene-modified T cells as immunotherapy for multiple myeloma and acute myeloid leukemia expressing the Lewis Y antigen. Gene Ther.

[100] Ritchie DS, Neeson PJ, Khot A, Peinert S, Tai T, Tainton K, Chen K, Shin M, Wall DM, Honemann D, Gambell P, Westerman DA, Haurat J, Westwood JA, Scott AM, Kravets L, Dickinson M, Trapani JA, Smyth MJ, Darcy PK, Kershaw MH, Prince HM (2013). Persistence and efficacy of second-generation CAR T cell against the LeY antigen in acute myeloid leukemia. Mol Ther J Am Soc Gene Ther.

[101] Lynn RC, Poussin M, Kalota A, Feng Y, Low PS, Dimitrov DS, Powell DJ (2015). Targeting of folate receptor β on acute myeloid leukemia blasts with chimeric antigen receptor–expressing T cells. Blood.

[102] John S, Chen H, Deng M, Gui X, Wu G, Chen W, Li Z, Zhang N, An Z, Zhang CC (2018). A novel anti-LILRB4 CAR-T cell for the treatment of monocytic AML. Mol Ther.

[103] Barber A, Meehan KR, Sentman CL (2011). Treatment of multiple myeloma with adoptively transferred chimeric NKG2D receptor-expressing T cells. Gene Ther.

[104] Barber A, Zhang T, Sentman CL (2008). Immunotherapy with chimeric NKG2D receptors leads to long-term tumor-free survival and development of host antitumor immunity in murine ovarian cancer. J Immunol.

[105] Ma Q, Garber HR, Lu S, He H, Tallis E, Ding X, Sergeeva A, Wood MS, Dotti G, Salvado B (2016). A novel TCR-like CAR with specificity for PR1/HLA-A2 effectively targets myeloid leukemia in vitro when expressed in human adult peripheral blood and cord blood T cells. Cytotherapy.

[106] Rafiq S, Purdon T, Daniyan A, Koneru M, Dao T, Liu C, Scheinberg D, Brentjens R (2017). Optimized T-cell receptor-mimic chimeric antigen receptor T cells directed toward the intracellular Wilms Tumor 1 antigen. Leukemia.

[107] Saxena A, Sheridan DP, Card RT, McPeek AM, Mewdell CC, Skinnider LF (1998). Biologic and clinical significance of CD7 expression in acute myeloid leukemia. Am J Hematol.

[108] Gomes-Silva D, Srinivasan M, Sharma S, Lee CM, Wagner DL, Davis TH, Rouce RH, Bao G, Brenner MK, Mamonkin M (2017). CD7-edited T cells expressing a CD7-specific CAR for the therapy of T-cell malignancies. Blood.

[109] Laszlo GS, Estey EH, Walter RB (2014). The past and future of CD33 as therapeutic target in acute myeloid leukemia. Blood Rev.

[110] Walter RB, Gooley TA, Van Der Velden VH, Loken MR, Van Dongen JJ, Flowers DA, Bernstein ID, Appelbaum FR (2007). CD33 expression and P-glycoprotein–mediated drug efflux inversely correlate and predict clinical outcome in patients with acute myeloid leukemia treated with gemtuzumab ozogamicin monotherapy. Blood.

[111] Dutour A, Marin V, Pizzitola I, Valsesia-Wittmann S, Lee D, Yvon E, et al. In vitro and in vivo antitumor effect of anti-cd33 chimeric receptor-expressing EBV-CTL against acute myeloid leukemia. Adv Hematol. 2012;2012. 10.1155/2012/683065 PMC3261457 22272203

[112] Marin V, Pizzitola I, Agostoni V, Attianese GMPG, Finney H, Lawson A, Pule M, Rousseau R, Biondi A, Biagi E (2010). Cytokine-induced killer cells for cell therapy of acute myeloid leukemia: improvement of their immune activity by expression of CD33-specific chimeric receptors. Haematologica.

[113] Wang Q-s, Wang Y, Lv H-y, Han Q-w, Fan H, Guo B, Wang L-l, Han W-d (2015). Treatment of CD33-directed chimeric antigen receptor-modified T cells in one patient with relapsed and refractory acute myeloid leukemia. Mol Ther.

[114] Kim MY, Yu K-R, Kenderian SS, Ruella M, Chen S, Shin T-H, Aljanahi AA, Schreeder D, Klichinsky M, Shestova O (2018). Genetic inactivation of CD33 in hematopoietic stem cells to enable CAR T cell immunotherapy for acute myeloid leukemia. Cell.

[115] Konopleva M, Rissling I, Andreeff M (2000). CD38 in hematopoietic malignancies. Chem Immunol.

[116] Mehta K, Ocanas L, Malavasi F, Marks JW, Rosenblum MG (2004). Retinoic acid-induced CD38 antigen as a target for immunotoxin-mediated killing of leukemia cells. Mol Cancer Ther.

[117] Günthert U, Hofmann M, Rudy W, Reber S, Zöller M, Hauβmann I, Matzku S, Wenzel A, Ponta H, Herrlich P (1991). A new variant of glycoprotein CD44 confers metastatic potential to rat carcinoma cells. Cell.

[118] Legras S, Günthert U, Stauder R, Curt F, Oliferenko S, Kluin-Nelemans H, Marie J, Proctor S, Jasmin C, Smadja-Joffe F (1998). A strong expression of CD44-6v correlates with shorter survival of patients with acute myeloid leukemia. Blood.

[119] Neu S, Geiselhart A, Sproll M, Hahn D, Kuci S, Niethammer D, Handgretinger R (1997). Expression of CD44 isoforms by highly enriched CD34-positive cells in cord blood, bone marrow and leukaphereses. Bone Marrow Transplant.

[120] Ciceri F, Bonini C, Stanghellini MTL, Bondanza A, Traversari C, Salomoni M, Turchetto L, Colombi S, Bernardi M, Peccatori J (2009). Infusion of suicide-gene-engineered donor lymphocytes after family haploidentical haemopoietic stem-cell transplantation for leukaemia (the TK007 trial): a non-randomised phase I–II study. Lancet Oncol.

[121] Di Stasi A, Tey S-K, Dotti G, Fujita Y, Kennedy-Nasser A, Martinez C, Straathof K, Liu E, Durett AG, Grilley B (2011). Inducible apoptosis as a safety switch for adoptive cell therapy. N Engl J Med.

[122] Jordan C, Upchurch D, Szilvassy S, Guzman M, Howard D, Pettigrew A, Meyerrose T, Rossi R, Grimes B, Rizzieri D (2000). The interleukin-3 receptor alpha chain is a unique marker for human acute myelogenous leukemia stem cells. Leukemia.

[123] Frankel A, Liu J-S, Rizzieri D, Hogge D (2008). Phase I clinical study of diphtheria toxin-interleukin 3 fusion protein in patients with acute myeloid leukemia and myelodysplasia. Leuk Lymphoma.

[124] Thokala R, Olivares S, Mi T, Maiti S, Deniger D, Huls H, Torikai H, Singh H, Champlin RE, Laskowski T (2016). Redirecting specificity of T cells using the sleeping beauty system to express chimeric antigen receptors by mix-and-matching of VL and VH domains targeting CD123+ tumors. PLoS One.

[125] Kottaridis PD, Gale RE, Frew ME, Harrison G, Langabeer SE, Belton AA, Walker H, Wheatley K, Bowen DT, Burnett AK (2001). The presence of a FLT3 internal tandem duplication in patients with acute myeloid leukemia (AML) adds important prognostic information to cytogenetic risk group and response to the first cycle of chemotherapy: analysis of 854 patients from the United Kingdom Medical Research Council AML 10 and 12 trials. Blood.

[126] Gilliland DG, Griffin JD (2002). The roles of FLT3 in hematopoiesis and leukemia, Blood. J Am Soc Hematol.

[127] Yamamoto Y, Kiyoi H, Nakano Y, Suzuki R, Kodera Y, Miyawaki S, Asou N, Kuriyama K, Yagasaki F, Shimazaki C (2001). Activating mutation of D835 within the activation loop of FLT3 in human hematologic malignancies. Blood.

[128] Takahashi S (2011). Downstream molecular pathways of FLT3 in the pathogenesis of acute myeloid leukemia: biology and therapeutic implications. J Hematol Oncol.

[129] Döhner H, Weisdorf DJ, Bloomfield CD (2015). Acute myeloid leukemia. N Engl J Med.

[130] Wang Y, Xu Y, Li S, Liu J, Xing Y, Xing H, Tian Z, Tang K, Rao Q, Wang M (2018). Targeting FLT3 in acute myeloid leukemia using ligand-based chimeric antigen receptor-engineered T cells. J Hematol Oncol.

[131] Van Rhenen A, Van Dongen GA, Kelder A, Rombouts EJ, Feller N, Moshaver B, Stigter-van Walsum M, Zweegman S, Ossenkoppele GJ, Schuurhuis GJ (2007). The novel AML stem cell–associated antigen CLL-1 aids in discrimination between normal and leukemic stem cells. Blood.

[132] Wiersma VR, de Bruyn M, Shi C, Gooden MJ, Wouters MC, Samplonius DF, Hendriks D, Nijman HW, Wei Y, Zhou J (2015). C-type lectin-like molecule-1 (CLL1)-targeted TRAIL augments the tumoricidal activity of granulocytes and potentiates therapeutic antibody-dependent cell-mediated cytotoxicity. MAbs, Taylor & Francis.

[133] Westwood JA, Murray WK, Trivett M, Haynes NM, Solomon B, Mileshkin L, Ball D, Michael M, Burman A, Mayura-Guru P (2009). The Lewis-Y carbohydrate antigen is expressed by many human tumors and can serve as a target for genetically redirected T cells despite the presence of soluble antigen in serum. J Immunother.

[134] Tashiro H, Sauer T, Shum T, Parikh K, Mamonkin M, Omer B, Rouce RH, Lulla P, Rooney CM, Gottschalk S (2017). Treatment of acute myeloid leukemia with T cells expressing chimeric antigen receptors directed to C-type lectin-like molecule 1. Mol Ther.

[135] Neeson P, Shin A, Tainton K, Guru P, Prince H, Harrison S, Peinert S, Smyth M, Trapani J, Kershaw M (2010). Ex vivo culture of chimeric antigen receptor T cells generates functional CD8+ T cells with effector and central memory-like phenotype. Gene Ther.

[136] Ross JF, Wang H, Behm FG, Mathew P, Wu M, Booth R, Ratnam M (1999). Folate receptor type β is a neutrophilic lineage marker and is differentially expressed in myeloid leukemia. Cancer Interdiscip Int J Am Cancer Soc.

[137] Pan XQ, Zheng X, Shi G, Wang H, Ratnam M, Lee RJ (2002). Strategy for the treatment of acute myelogenous leukemia based on folate receptor β–targeted liposomal doxorubicin combined with receptor induction using all-trans retinoic acid. Blood.

[138] Wang H, Zheng X, Behm FG, Ratnam M (2000). Differentiation-independent retinoid induction of folate receptor type β, a potential tumor target in myeloid leukemia. Blood.

[139] Dobrowolska H, Gill KZ, Serban G, Ivan E, Li Q, Qiao P, Suciu-Foca N, Savage D, Alobeid B, Bhagat G (2013). Expression of immune inhibitory receptor ILT3 in acute myeloid leukemia with monocytic differentiation. Cytom Part B Clin Cytom.

[140] Garrity D, Call ME, Feng J, Wucherpfennig KW (2005). The activating NKG2D receptor assembles in the membrane with two signaling dimers into a hexameric structure. Proc Natl Acad Sci.

[141] Lanier LL (2015). NKG2D receptor and its ligands in host defense. Cancer Immunol Res.

[142] Spear P, Barber A, Rynda-Apple A, Sentman CL (2013). NKG2D CAR T-cell therapy inhibits the growth of NKG2D ligand heterogeneous tumors. Immunol Cell Biol.

[143] Spear P, Wu M-R, Sentman M-L, Sentman CL (2013). NKG2D ligands as therapeutic targets. Cancer Immun Arch.

[144] Zhang T, Barber A, Sentman CL (2007). Chimeric NKG2D–modified T cells inhibit systemic T-cell lymphoma growth in a manner involving multiple cytokines and cytotoxic pathways. Cancer Res.

[145] Baumeister SH, Murad J, Werner L, Daley H, Trebeden-Negre H, Gicobi JK, et al. Phase 1 trial of autologous CAR T cells targeting NKG2D ligands in patients with AML/MDS and multiple myeloma. Cancer Immunol Res. 2018. 10.1158/2326-6066.CIR-18-0307 PMC7814996 30396908

[146] Molldrem JJ, Clave E, Jiang YZ, Mavroudis D, Raptis A, Hensel N, Agarwala V, Barrett AJ (1997). Cytotoxic T lymphocytes specific for a nonpolymorphic proteinase 3 peptide preferentially inhibit chronic myeloid leukemia colony-forming units. Blood.

[147] Di Stasi A, Jimenez AM, Minagawa K, Al-Obaidi M, Rezvani K (2015). Review of the results of WT1 peptide vaccination strategies for myelodysplastic syndromes and acute myeloid leukemia from nine different studies. Front Immunol.

[148] Morrison AA, Viney RL, Ladomery MR. The post-transcriptional roles of WT1, a multifunctional zinc-finger protein, Biochimica Et Biophysica Acta (BBA)-Reviews on Cancer. 2008;1785:55–62. 10.1016/j.bbcan.2007.10.002 17980713

[149] Sugiyama H (2010). WT1 (Wilms' tumor gene 1): biology and cancer immunotherapy. Japanese J Clin Oncol.

[150] Brudno JN, Kochenderfer JN (2018). Chimeric antigen receptor T-cell therapies for lymphoma. Nat Rev Clin Oncol.

[151] Garfall AL, Maus MV, Hwang W-T, Lacey SF, Mahnke YD, Melenhorst JJ, Zheng Z, Vogl DT, Cohen AD, Weiss BM (2015). Chimeric antigen receptor T cells against CD19 for multiple myeloma. N Engl J Med.

[152] Kershaw MH, Westwood JA, Parker LL, Wang G, Eshhar Z, Mavroukakis SA, White DE, Wunderlich JR, Canevari S, Rogers-Freezer L (2006). A phase I study on adoptive immunotherapy using gene-modified T cells for ovarian cancer. Clin Cancer Res.

[153] Newick K, O'Brien S, Moon E, Albelda SM (2017). CAR T cell therapy for solid tumors. Annu Rev Med.

[154] Brown CE, Alizadeh D, Starr R, Weng L, Wagner JR, Naranjo A, Ostberg JR, Blanchard MS, Kilpatrick J, Simpson J (2016). Regression of glioblastoma after chimeric antigen receptor T-cell therapy. N Engl J Med.

[155] Butler MO, Hirano N (2014). Human cell-based artificial antigen-presenting cells for cancer immunotherapy. Immunol Rev.

[156] Xu Y, Zhang M, Ramos CA, Durett A, Liu E, Dakhova O, Liu H, Creighton CJ, Gee AP, Heslop HE (2014). Closely related T-memory stem cells correlate with in vivo expansion of CAR. CD19-T cells and are preserved by IL-7 and IL-15. Blood.

[157] Gattinoni L, Klebanoff CA, Palmer DC, Wrzesinski C, Kerstann K, Yu Z, Finkelstein SE, Theoret MR, Rosenberg SA, Restifo NP (2005). Acquisition of full effector function in vitro paradoxically impairs the in vivo antitumor efficacy of adoptively transferred CD8+ T cells. J Clin Invest.

[158] Hinrichs CS, Borman ZA, Cassard L, Gattinoni L, Spolski R, Yu Z, Sanchez-Perez L, Muranski P, Kern SJ, Logun C (2009). Adoptively transferred effector cells derived from naive rather than central memory CD8+ T cells mediate superior antitumor immunity. Proc Natl Acad Sci.

[159] Radvanyi LG, Bernatchez C, Zhang M, Fox PS, Miller P, Chacon J, Wu R, Lizee G, Mahoney S, Alvarado G (2012). Specific lymphocyte subsets predict response to adoptive cell therapy using expanded autologous tumor-infiltrating lymphocytes in metastatic melanoma patients. Clin Cancer Res.

[160] Kowolik CM, Topp MS, Gonzalez S, Pfeiffer T, Olivares S, Gonzalez N, Smith DD, Forman SJ, Jensen MC, Cooper LJ (2006). CD28 costimulation provided through a CD19-specific chimeric antigen receptor enhances in vivo persistence and antitumor efficacy of adoptively transferred T cells. Cancer Res.

[161] Long AH, Haso WM, Shern JF, Wanhainen KM, Murgai M, Ingaramo M, Smith JP, Walker AJ, Kohler ME, Venkateshwara VR (2015). 4-1BB costimulation ameliorates T cell exhaustion induced by tonic signaling of chimeric antigen receptors. Nat Med.

[162] Song D-G, Ye Q, Poussin M, Harms GM, Figini M, Powell DJ (2012). CD27 costimulation augments the survival and antitumor activity of redirected human T cells in vivo. Blood.

[163] Lai Y, Weng J, Wei X, Qin L, Lai P, Zhao R, Jiang Z, Li B, Lin S, Wang S (2018). Toll-like receptor 2 costimulation potentiates the antitumor efficacy of CAR T Cells. Leukemia.

[164] Mata M, Gerken C, Nguyen P, Krenciute G, Spencer DM, Gottschalk S (2017). Inducible activation of MyD88 and CD40 in CAR T cells results in controllable and potent antitumor activity in preclinical solid tumor models. Cancer Discov.

[165] Maude SL, Laetsch TW, Buechner J, Rives S, Boyer M, Bittencourt H, Bader P, Verneris MR, Stefanski HE, Myers GD (2018). Tisagenlecleucel in children and young adults with B-cell lymphoblastic leukemia. N Engl J Med.

[166] Ruella M, Xu J, Barrett DM, Fraietta JA, Reich TJ, Ambrose DE, Klichinsky M, Shestova O, Patel PR, Kulikovskaya I (2018). Induction of resistance to chimeric antigen receptor T cell therapy by transduction of a single leukemic B cell. Nat Med.

[167] Shalabi H, Kraft IL, Wang H-W, Yuan CM, Yates B, Delbrook C, Zimbelman JD, Giller R, Stetler-Stevenson M, Jaffe ES (2018). Sequential loss of tumor surface antigens following chimeric antigen receptor T-cell therapies in diffuse large B-cell lymphoma. Haematologica.

[168] Ren J, Zhao Y (2017). Advancing chimeric antigen receptor T cell therapy with CRISPR/Cas9. Protein Cell.

[169] Brudno JN, Somerville RP, Shi V, Rose JJ, Halverson DC, Fowler DH, Gea-Banacloche JC, Pavletic SZ, Hickstein DD, Lu TL (2016). Allogeneic T cells that express an anti-CD19 chimeric antigen receptor induce remissions of B-cell malignancies that progress after allogeneic hematopoietic stem-cell transplantation without causing graft-versus-host disease. J Clin Oncol.

[170] Austin R, Smyth MJ, Lane SW (2016). Harnessing the immune system in acute myeloid leukaemia. Crit Rev Oncol Hematol.

[171] Teague RM, Kline J (2013). Immune evasion in acute myeloid leukemia: current concepts and future directions. J Immunother Cancer.

[172] Vago L, Perna SK, Zanussi M, Mazzi B, Barlassina C, Stanghellini MTL, Perrelli NF, Cosentino C, Torri F, Angius A (2009). Loss of mismatched HLA in leukemia after stem-cell transplantation. N Engl J Med.

[173] Kochenderfer JN, Dudley ME, Feldman SA, Wilson WH, Spaner DE, Maric I, Stetler-Stevenson M, Phan GQ, Hughes MS, Sherry RM (2012). B-cell depletion and remissions of malignancy along with cytokine-associated toxicity in a clinical trial of anti-CD19 chimeric-antigen-receptor–transduced T cells. Blood.

[174] Shimabukuro-Vornhagen A, Gödel P, Subklewe M, Stemmler HJ, Schlößer HA, Schlaak M, Kochanek M, Böll B, von Bergwelt-Baildon MS (2018). Cytokine release syndrome. J Immunother Cancer.

[175] Brentjens RJ, Rivière I, Park JH, Davila ML, Wang X, Stefanski J, Taylor C, Yeh R, Bartido S, Borquez-Ojeda O (2011). Safety and persistence of adoptively transferred autologous CD19-targeted T cells in patients with relapsed or chemotherapy refractory B-cell leukemias. Blood.

[176] Beatty GL, Haas AR, Maus MV, Torigian DA, Soulen MC, Plesa G, Chew A, Zhao Y, Levine BL, Albelda SM (2014). Mesothelin-specific chimeric antigen receptor mRNA-engineered T cells induce antitumor activity in solid malignancies. Cancer Immunol Res.

[177] Morgan RA, Yang JC, Kitano M, Dudley ME, Laurencot CM, Rosenberg SA (2010). Case report of a serious adverse event following the administration of T cells transduced with a chimeric antigen receptor recognizing ERBB2. Mol Ther.

[178] Lamers CH, Langeveld SC, Groot-van Ruijven CM, Debets R, Sleijfer S, Gratama JW (2007). Gene-modified T cells for adoptive immunotherapy of renal cell cancer maintain transgene-specific immune functions in vivo. Cancer Immunol Immunother.

[179] Davila ML, Brentjens RJ (2016). CD19-Targeted CAR T cells as novel cancer immunotherapy for relapsed or refractory B-cell acute lymphoblastic leukemia. Clin Adv Hematol Oncol.

[180] Liu X, Zhang N, Shi H. Driving better and safer HER2-specific CARs for cancer therapy. Oncotarget. 2017;8(37):62730. 10.18632/oncotarget.17528. 10.18632/oncotarget.17528 PMC5617544 28977984

[181] Maus MV, Haas AR, Beatty GL, Albelda SM, Levine BL, Liu X, Zhao Y, Kalos M, June CH (2013). T cells expressing chimeric antigen receptors can cause anaphylaxis in humans. Cancer Immunol Res.

[182] Bonifant CL, Jackson HJ, Brentjens RJ, Curran KJ (2016). Toxicity and management in CAR T-cell therapy. Mol Thery Oncolytics.

[183] Scholler J, Brady TL, Binder-Scholl G, Hwang W-T, Plesa G, Hege KM, Vogel AN, Kalos M, Riley JL, Deeks SG (2012). Decade-long safety and function of retroviral-modified chimeric antigen receptor T cells. Sci Transl Med.

[184] Fried S, Avigdor A, Bielorai B, Meir A, Besser MJ, Schachter J, et al. Early and late hematologic toxicity following CD19 CAR-T cells. Bone Marrow Transplant. 2019;1. 10.1038/s41409-019-0487-3 30809033

[185] Laszlo GS, Harrington KH, Gudgeon CJ, Beddoe ME, Fitzgibbon MP, Ries RE, Lamba JK, McIntosh MW, Meshinchi S, Walter RB (2016). Expression and functional characterization of CD33 transcript variants in human acute myeloid leukemia. Oncotarget.

[186] Yáñez L, Sánchez-Escamilla M, Perales M-A (2019). CAR T cell toxicity: current management and future directions. HemaSphere.

[187] Lee DW, Gardner R, Porter DL, Louis CU, Ahmed N, Jensen M, Grupp SA, Mackall CL (2014). Current concepts in the diagnosis and management of cytokine release syndrome. Blood.

[188] Norelli M, Camisa B, Barbiera G, Falcone L, Purevdorj A, Genua M, Sanvito F, Ponzoni M, Doglioni C, Cristofori P (2018). Monocyte-derived IL-1 and IL-6 are differentially required for cytokine-release syndrome and neurotoxicity due to CAR T cells. Nat Med.

[189] Ruella M, Kenderian SS, Shestova O, Klichinsky M, Melenhorst JJ, Wasik MA, et al. Kinase inhibitor ibrutinib prevents cytokine-release syndrome after anti-CD19 chimeric antigen receptor T cells (CART) for B cell neoplasms. London: In: Am Soc Hematology; 2016. 10.1038/leu.2016.262 27677739

[190] Giavridis T, van der Stegen SJ, Eyquem J, Hamieh M, Piersigilli A, Sadelain M (2018). CAR T cell–induced cytokine release syndrome is mediated by macrophages and abated by IL-1 blockade. Nat Med.

[191] Sterner RM, Sakemura R, Cox MJ, Yang N, Khadka RH, Forsman CL, Hansen MJ, Jin F, Ayasoufi K, Hefazi M (2019). GM-CSF inhibition reduces cytokine release syndrome and neuroinflammation but enhances CAR-T cell function in xenografts. Blood.

[192] Maude SL, Barrett DM, Ambrose DE, Rheingold SR, Aplenc R, Teachey DT, Callahan C, Barker CS, Mudambi M, Shaw PA (2015). Efficacy and safety of humanized chimeric antigen receptor (CAR)-modified T cells targeting CD19 in children with relapsed/refractory ALL. Am Soc Hematology.

[193] Cummins KD, Gill S (2019). Will CAR T cell therapy have a role in AML? Promises and pitfalls. Seminars in hematology.

[194] Tiberghien P, Cahn J-Y, Brion A, Deconinck E, Racadot E, Hervé P, Milpied N, Lioure B, Gluckman E, Bordigoni P (1997). Use of donor T-lymphocytes expressing herpes-simplex thymidine kinase in allogeneic bone marrow transplantation: a phase I–II study. Lab d'Histocompatibilité Thér Immuno Mol Besançon Fr Hum Gene Ther.

[195] Tey S-K, Dotti G, Rooney CM, Heslop HE, Brenner MK (2007). Inducible caspase 9 suicide gene to improve the safety of allodepleted T cells after haploidentical stem cell transplantation. Biol Blood Marrow Transplant.

[196] Thomis DC, Marktel S, Bonini C, Traversari C, Gilman M, Bordignon C, Clackson T (2001). A Fas-based suicide switch in human T cells for the treatment of graft-versus-host disease. Blood.

[197] Philip B, Kokalaki E, Mekkaoui L, Thomas S, Straathof K, Flutter B, Marin V, Marafioti T, Chakraverty R, Linch D (2014). A highly compact epitope-based marker/suicide gene for easier and safer T-cell therapy. Blood.

[198] Serafini M, Manganini M, Borleri G, Bonamino M, Imberti L, Biondi A, Golay J, Rambaldi A, Introna M (2004). Characterization of CD20-transduced T lymphocytes as an alternative suicide gene therapy approach for the treatment of graft-versus-host disease. Hum Gene Ther.

[199] Li H, Zhao Y (2017). Increasing the safety and efficacy of chimeric antigen receptor T cell therapy. Protein Cell.

[200] Perna F, Berman SH, Soni RK, Mansilla-Soto J, Eyquem J, Hamieh M, Hendrickson RC, Brennan CW, Sadelain M (2017). Integrating proteomics and transcriptomics for systematic combinatorial chimeric antigen receptor therapy of AML. Cancer Cell.

[201] Cartellieri M, Feldmann A, Koristka S, Arndt C, Loff S, Ehninger AV, Von Bonin M, Bejestani E, Ehninger G, Bachmann M (2016). Switching CAR T cells on and off: a novel modular platform for retargeting of T cells to AML blasts. Blood Cancer J.

